# Patient-Derived Cells to Guide Targeted Therapy for Advanced Lung Adenocarcinoma

**DOI:** 10.1038/s41598-019-56356-4

**Published:** 2019-12-27

**Authors:** Seok-Young Kim, Ji Yeon Lee, Dong Hwi Kim, Hyeong -Seok Joo, Mi Ran Yun, Dongmin Jung, Jiyeon Yun, Seong Gu Heo, Beung -Chul Ahn, Chae Won Park, Kyoung Ho Pyo, You Jin Chun, Min Hee Hong, Hye Ryun Kim, Byoung Chul Cho

**Affiliations:** 1 JEUK Institute for Cancer Research, JEUK Co.,Ltd., Gumi-City, Kyungbuk Korea; 2 Yonsei Cancer Center, Division of Medical Oncology, Department of Internal Medicine, Yonsei University College of Medicine, Seoul, Korea; 3 Severance Biomedical Science Institute, Yonsei University of College of Medicine, Seoul, Korea

**Keywords:** Cancer models, Cancer models, Targeted therapies, Non-small-cell lung cancer

## Abstract

Adequate preclinical model and model establishment procedure are required to accelerate translational research in lung cancer. We streamlined a protocol for establishing patient-derived cells (PDC) and identified effective targeted therapies and novel resistance mechanisms using PDCs. We generated 23 PDCs from 96 malignant effusions of 77 patients with advanced lung adenocarcinoma. Clinical and experimental factors were reviewed to identify determinants for PDC establishment. PDCs were characterized by driver mutations and *in vitro* sensitivity to targeted therapies. Seven PDCs were analyzed by whole-exome sequencing. PDCs were established at a success rate of 24.0%. Utilizing cytological diagnosis and tumor colony formation can improve the success rate upto 48.8%. *In vitro* response to a tyrosine kinase inhibitor (TKI) in PDC reflected patient treatment response and contributed to identifying effective therapies. Combination of dabrafenib and trametinib was potent against a rare *BRAF* K601E mutation. Afatinib was the most potent EGFR-TKI against uncommon *EGFR* mutations including L861Q, G719C/S768I, and D770_N771insG. Aurora kinase A (AURKA) was identified as a novel resistance mechanism to olmutinib, a mutant-selective, third-generation EGFR-TKI, and inhibition of AURKA overcame the resistance. We presented an efficient protocol for establishing PDCs. PDCs empowered precision medicine with promising translational values.

## Introduction

Non-small-cell lung cancer (NSCLC) is a leading cause of cancer-related deaths worldwide. Oncogenic driver mutations have been identified in NSCLC including epidermal growth factor receptor gene (*EGFR*) mutations, anaplastic lymphoma kinase gene (*ALK*) fusions, v-raf murine sarcoma viral oncogene homolog B (*BRAF*) mutations, and *ROS1* fusions. Over the last decade, small molecule tyrosine kinase inhibitors (TKI) have been developed to target these mutations, which revolutionized therapeutic landscape in NSCLC; Treatment with TKIs have prolonged survival and increased disease control in patients with advanced NSCLC^1,2^.

Unfortunately, most patients eventually relapse within a year on TKI therapy. To date, various mechanisms of acquired resistance to TKIs have been reported. The most common molecular mechanisms of resistance are secondary mutations in kinase domains of the drug targets and activation of alternative pathways^3–5^. With advances in molecular profiling of acquired resistance, new therapeutic strategies, such as combination targeted therapies and next-generation TKIs, have been introduced to overcome the TKI resistance^1^. On the other hand, molecular determinants that clearly guide subsequent therapy have not been observed in some patients who failed to previous treatment.

Drug-resistant cell lines that are established following chronic exposure to a drug *in vitro* are conventionally used for studying the mechanisms of TKI resistance in NSCLC. However, a limited panel of NSCLC cell lines harboring the *EGFR* mutation, *ALK* fusion, or *ROS1* fusion is commercially-available. Additionally, these models may exhibit different patterns of drug sensitivity likely due to lack of genetic complexity found in patients^6^.

Patient-derived cells (PDC) generated from tumor specimens have shown to reflect patient tumor characteristics and clinical responses^7^. The practical challenges for primary culture of tumor cells involve limited availability of tumor specimens, outgrowth of stromal cells, and tumor cell senescence^8,9^. Here, we evaluated clinical and experimental factors that may impact a success rate of PDC establishment, which can accelerate model establishment procedure and promote translational research. We also investigated resistance mechanisms and novel combinational therapies to overcome resistance to third-generation EGFR-TKIs in *EGFR*-mutant NSCLC using our PDCs.

## Materials and Methods

### Ethics approval

This study was approved by Yonsei University Hospital Institutional Review Board (Seoul, Korea) (IRB no.: 4-2016-0788). This study was conducted according to the Declaration of Helsinki and all patients provided informed consent.

### Sample processing and establishment of patient-derived cells

A total of 96 malignant effusions (89 pleural effusions, 4 pericardial effusions, and 3 ascites) were collected from 77 patients with advanced lung adenocarcinoma at Yonsei Cancer Center between March 2016 and July 2018. Fluorescence *in situ* hybridization, immunohistochemistry, and direct sequencing were routinely performed for initial diagnosis of lung adenocarcinoma. PANAMutyper^TM^R (Panagene, Daejeon, Korea) was routinely performed for genotyping of *EGFR*-mutant NSCLC at recurrence. Clinical data were retrieved based on electric medical record system.

All samples were kept on ice during transport from Yonsei Cancer Center to the laboratory where samples were processed. Ninety-two cases were assessed for malignancy by a pathologist (HS Shim), whereas four cases were rejected due to insufficient amount of cells. Samples that were positive for malignancy were defined as M+. Otherwise, samples were defined as M−.

Malignant effusions were processed as previously described^10,11^. In brief, samples were centrifuged at 500 g for 10 min before cell pellets were suspended in PBS. Then, cells were separated by density gradient centrifugation using Ficoll-PaquePLUS (GE Healthecare Bio-Sciences, Uppsala, Sweden). Mononuclear cells including tumor cells were isolated from the interphase layer, washed twice with HBSS, suspended in R10 medium [RPMI-1640 with 10% fetal bovine serum, 5% penicillin/streptomycin, and 1% primocin (Invitrogen, Massachusetts, USA)], and seeded on collagen IV pre-coated culture plates at a density of approximately 1 to 2 × 10^6^ cells per plate. After culture initiation, cells were observed for 10 days using light microscopy. Tumor colony formation, defined by multiple lung adenocarcinoma cells (n ≥ 2) adherent to a culture plate, was occasionally observed in primary cultures (Supplementary Fig. 1)^10^. If such tumor colony formation was detected, a primary culture was defined as TCF+. Otherwise, a primary culture was defined as TCF- (Supplementary Fig. 1). Primary cultures were frequently contaminated with stromal cells (Supplementary Table 1). To achieve high tumor purity, differential trypsinization was regularly performed (Supplementary Fig. 2)^7,12^.

### Cell culture

H2291 cells were obtained from the American Type Culture Collection and MRC-5 cells were obtained from Korean Cell Line Bank. PC9 cells were provided by J.C. Lee (Korea Institute of Radiological and Medical Science, Seoul, Korea). H2291, PC9, and all PDCs were cultured in R10 medium. MRC-5 cells were cultured in MEM supplemented with 10% fetal bovine serum, 5% penicillin/streptomycin, and 1% primocin. All cells were replenished with medium every three days and maintained in a 5% CO2 incubator at 37 °C. Cells were passaged at ratios of 1:2 to 1:6 using TrypLE (Invitrogen).

### Flow cytometry

Cells were stained with a human EpCAM antibody or a human fibroblast antibody (Miltenyi Biotec, Bergisch Gladbach, Germany) according to the manufacturer’s instructions. Flow cytometry was performed using FACSVerse (BD Biosciences, California, USA) and analyzed with FlowJo software.

### DNA and RNA extractions

gDNA was extracted from PDCs using the DNeasy Blood & Tissue Kits (Qiagen, Venlo, Netherlands). In 5 cases (YU-1088, YU-1094, YU-1095, YU-1096, and YU-1097), germline DNA was extracted from normal blood of corresponding patients using the DNeasy Blood & Tissue Kits (Qiagen). RNA was extracted from PDCs using TRIzol (Invitrogen) before being synthesized to cDNA using SuperScript III First-Strand Synthesis System.

### Direct sequencing

*EGFR* sequencing service was provided by Macrogen Inc. (Seoul, Korea). *EML4-ALK* gene arrangements were PCR amplified as previously described^13^. *CD74-ROS1, TPM3-ROS1*, *SLC34A2-ROS1* genes were PCR amplified using AccuPower^®^ PCR Premix (Bioneer, Seoul, Korea). All PCR primers used in this study are listed in Supplementary Table 2.

### Whole-exome sequencing and data analysis

gDNA purity and concentration were tested by PicoGreen^®^ dsDNA assay (Invitrogen) and agarose gel electrophoresis method. Genomic fragment library was prepared using SureSelect v5 Kit (Agilent Technologies, Santa Clara, CA) and then sequenced on Illumina HiSeq 2500 (California, USA). The resulting sequencing reads were mapped to the human genome reference (hg19) using the Burrows-Wheeler alignment tool^14,15^. Somatic mutations were called using MuTect2. In 2 cases (YU-1070 and YU-1089) which lack corresponding normal blood samples, germline variants were filtered out using ExAC_AF database at a frequency of >0.01. Copy number variation was analyzed by CNVkit in PDCs (YU-1088, YU-1094, YU-1095, YU-1096, and YU-1097) where corresponding normal blood samples were available^16^. Annotation was performed with cosmic database^17,18^.

### Cell viability assays

Cells were seeded at a density of 2500–5000 per well in 96-well clear bottom microplates. Cells were incubated overnight and treated with drugs for 3 days. Cell viability was analyzed using CellTiter-Glo (Promega, Wisconsin, USA). IC_50_ values were calculated using GraphPad Prism version 5. Drugs used in the assays were purchased from Selleckchem (Texas, USA). Combination index (CI) was calculated using the Chou-Talalay method and the Bliss independence model^19,20^. For crystal violet assays, cells were seeded at a density of 20000 cells per well on 6-well plates. Cells were incubated overnight and exposed to the indicated drugs for 14 days. Medium containing drugs were replenished every 3 days.

### Immunoblot analysis

Bim, Bax, Cleaved PARP, BRAF, pCRAF (S338), CRAF, MEK, pMEK (S217/221), EGFR, pEGFR (Y1068), AKT, pAKT (S473), ERK, pERK (T202/Y204), AURKA, pAURKA, S6, pS6 (S240/244), and HRP–conjugated secondary antibodies were purchased from Cell Signaling Technology (Danvers, MA). Actin was obtained from Merck Millipore (Darmstadt, Germany). The immunoblots were detected by SuperSignal™ West Pico Chemiluminescent Substrate (Thermo Fisher Scientific, Massachusetts, USA).

### Statistical analysis

In univariate analysis, the Fisher’s exact test and Mann-Whitney U test were applied to investigate association between PDC establishment and variables. In multivariate analysis, multivariate logistic regression model was used.

## Results

### Positive cytological diagnosis of malignancy and tumor colony formation impact PDC establishment

A total of 23 PDCs were established from malignant effusions of advanced lung adenocarcinoma at a success rate of 24.0%. Established PDCs were free of stromal cells by light microscopy, strongly positive for EpCAM (an epithelial cell marker), could be frozen/thawed, and propagated at least 10 times (Supplementary Table 3 and Supplementary Fig. 3A)^7,21,22^.

**Figure 1:**
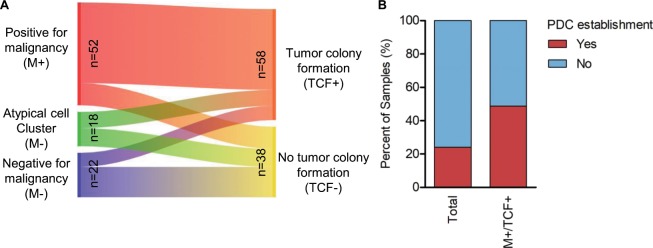
Factors critical to establishing PDCs of advanced lung adenocarcinoma. (**A**) Sankey plot visualizes correspondence between positive cytological diagnosis of malignancy (left) and tumor colony formation within 10 days of culture initiation (right) in malignant effusions. (**B**) Bar graph shows success rates of PDC establishment for total (n = 96) or M+/TCF+ samples (n = 41).

Previous studies have shown that several factors including genetic alteration impact success rate of patient-derived xenograft model establishment, whereas little is known about establishing PDC from advanced lung adenocarcinoma^23–25^. To address this question, we reviewed association of factors to PDC establishment. Univariate analysis revealed that positive cytological diagnosis of malignancy (M+) and tumor colony formation in the initial primary culture (TCF+) were strongly correlated with PDC establishment (OR = 8.3654, *P* < 0.001; OR = 22.0772, *P* < 0.001) as well as multivariate analysis (OR = 4.8336, *P* = 0.0239; OR = 14.1733, *P* = 0.0131) (Table 1 and Supplementary Fig. 1). As expected, high concordance was observed between M+ and TCF+ group in malignant effusions (Fig. 1A). The success rate was high in M+/TCF+subgroup (20/41, 48.8%), implying that these factors may be a powerful indicator of successful model establishment (Fig. 1B). A major reason for failure of model establishment was a paucity of tumor cells in samples (62/73; 84.9%) followed by tumor cell senescence (11/73; 15.1%).

**Table 1:** Univariable and multivariable analyses for determining factors correlated to PDC establishment.

Variate	PDC establishment		P^a^	OR (95% CI)
	No (n = 73)	Yes (n = 23)		
**Univariate analysis**				
Age	57 (19–88)	57 (19–77)	0.6031^b^	
Gender				0.7585 (0.2647–2.1520)
Female	40 (78.4%)	11 (21.6%)	0.6352	
Male	33 (73.3%)	12 (26.7%)		
Prior therapy				0.3005 (0.0206–4.3759)
Yes	71 (77.2%)	21 (22.8%)	0.2417	
No	2 (50.0%)	2 (50.0%)		
Prior chemotherapy				0.4745 (0.1626–1.3841)
Yes	51 (81.0%)	12 (19.0%)	0.1367	
No	22 (66.7%)	11 (33.3%)		
Prior TKI therapy				1.0289 (0.2727–4.8489)
Yes	60 (75.9%)	19 (24.1%)	1	
No	13 (76.5%)	4 (23.5%)		
EGFR mutation				0.5904 (0.1994–1.8049)
Yes	53 (79.1%)	14 (20.9%)	0.3064	
No	20 (69.0%)	9 (31.0%)		
ALK fusion				0.9455 (0.1524–4.1794)
Yes	10 (76.9%)	3 (23.1%)	1	
No	63 (75.9%)	20 (24.1%)		
ROS1 fusion				3.0575 (0.6588–13.6332)
Yes	6 (54.5%)	5 (45.5%)	0.1259	
No	67 (78.8%)	18 (21.2%)		
Source				1.9584 (0.2184–94.6450)
Pleural effusion	67 (75.3%)	22 (24.7%)	1	
Others	6 (85.7%)	1 (14.3%)		
Time to processing				1.9584 (0.2184–94.6450)
<4 hours	67 (75.3%)	22 (24.7%)	1	
>24 hours	6 (85.7%)	1 (14.3%)		
Sample volume	200 (10–1350)	200 (60–500)	0.58232^b^	
Cytology				8.3654 (2.1991–47.7354)
Malignancy	32 (61.5%)	20 (38.5%)	<0.001	
Others	41 (93.2%)	3 (6.8%)		
Tumor colony formation				22.0772 (3.2262–953.2819)
Yes	36 (62.1%)	22 (37.9%)	<0.001	
No	37 (97.4%)	1 (2.6%)		
**Multivariate analysis**^**c**^				
**Variate**			**P**	**OR (95% CI)**
Malignancy vs others			0.0239	4.8336 (1.2322–18.9608)
Tumor colony formation vs no colony formation			0.0131	14.1733 (1.7469–114.9934)

^a^The P value is calculated using the Fisher’s exact test unless indicated otherwise.

^b^The P value is calculated with the Mann-Whitney U test.

^c^The P value and odd ratio are analyzed by multivariate logistic regression.

PDC, patient-derived cells.

### Characteristics of PDCs

We characterized PDCs by direct sequencing (n = 23) and whole-exome sequencing (WES) (n = 7) (Table 2, Supplementary Table 4, and Supplementary Fig. 4). Fourteen *EGFR*-mutant cell lines were generated from *EGFR*-mutant tumors progressing to first- (n = 8), second- (n = 1), or third-generation EGFR-TKIs (n = 5). Routine genetic testing of rebiopsy samples at recurrence were available in 9 patients with *EGFR*-mutant NSCLC. Notably, *EGFR* genotypes detected in the rebiopsy samples were concordant to those in corresponding PDCs (Table 2). Three PDCs which were originated from ALK-positive NSCLC maintained *EML4-ALK* fusion genes. Five *ROS1*-fusion PDCs which were generated from ROS1-positive NSCLC maintained various *ROS1* fusion genes (*SLC34 A2-ROS1, CD74-ROS1*, and *TPM3-ROS1)* (Table 2). WES identified *BRAF K601E* as a driver mutation in YU-1070 cells that were derived from NSCLC without druggable genomic alterations (Supplementary Table 4 and Supplementary Fig. 4A). These results demonstrate that PDCs largely maintain known patient driver mutations.

**Table 2:** Characteristics of PDCs.

Cell line ID	Prior TKI therapy^b^	Driver mutation
YU-1073	Gefitinib	EGFR L858R/T790M^a^
YU-1074	Gefitinib	EGFR D770_N771insG
YU-1090	Gefitinib	EGFR L858R/T790M^a^
YU-1092	Gefitinib	EGFR L861Q
YU-1093	Erlotinib	EGFR exon 19 deletion^a^
YU-1094	Gefitinib	EGFR L858R^a^
YU-1099	Gefitinib	EGFR G719C/S768I^a^
YU-1152	Erlotinib	EGFR L858R^a^
YU-1091	Afatinib + Ruxolitinib	EGFR L858R^a^
YU-1088	Osimertinib	EGFR exon 19 deletion^a^
YU-1089	Olmutinib	EGFR exon 19 deletion^a^
YU-1095	Osimertinib	EGFR exon 19 deletion^a^
YU-1096	Osimertinib	EGFR L858R
YU-1097	Osimertinib	EGFR exon 19 deletion/T790M/C797S
YU-1080	N/A	CD74-ROS1
YU-1081	Crizotinib	TPM3-ROS1
YU-1082	N/A	SLC34A2-ROS1
YU-1083	N/A	SLC34A2-ROS1
YU-1085	Crizotinib	SLC34A2-ROS1
YU-1075	Crizotinib	EML4-ALK
YU-1076	Ceritinib	EML4-ALK
YU-1077	Alectinib	EML4-ALK G1202R
YU-1070	N/A	BRAF K601E

^a^Routine genetic testing results were available in re-biopsy samples after disease progression in these cases and confirmed driver mutations detected in corresponding PDCs.

^b^PDC was generated from advanced lung adenocarcinoma progressing to the annotated therapy.

N/A, not available.

Extensive passaging may result in a genetic drift of cell lines^26,27^. To investigate this issue, we analyzed 5 PDCs at early and later passages using direct sequencing (YU-1092, YU-1096, YU-1152, and YU-1097) or WES (YU-1094). A mutation allele frequency (MAF) of EGFR mutations were preserved upto approximately 30 passages (Supplementary Fig. 4B). Furthermore, somatic mutations and copy number variations were stably maintained between passages (Supplementary Fig. 4A,C, and D). These results may suggest that driver mutations and tumor-related genes are stably maintained at least in tested PDCs.

**Figure 2:**
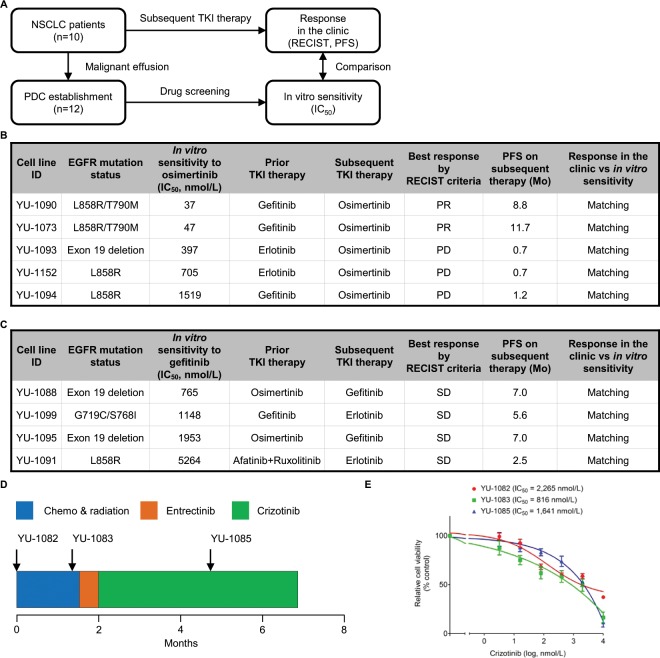
PDC modeling of patient treatment response to TKIs. (**A**) Twelve PDCs were established from 10 patients who received subsequent TKI therapy. Clinical follow-up data were retrospectively collected. At the same time, PDCs were screened with TKI which the patients received. *In vitro* sensitivity to the TKI in the PDC and response in the clinic were compared. (**B**) Summary of 5 EGFR-mutant PDCs which were generated from patients treated with osimertinib as subsequent TKI therapy. (**C**) Summary of 4 EGFR-mutant PDCs which were generated from patients treated with a first-generation EGFR-TKI as subsequent TKI therapy. (**D**) Clinical course of a patient with ROS1-positive NSCLC. YU-1082, YU-1083, and YU-1085 cells were derived from patient tumors at the indicated time points. (**E**) YU-1082, YU-1083, and 1085 cells were treated with the indicated concentrations of crizotinib. Data are presented as the mean ± SEM (n = 3). PR, partial response; PD, progressive-disease; SD, stable disease; PFS, progression-free survival.

Next, we compared *in vitro* sensitivity to TKI in PDC with response in the clinic. Ten patients in our study received subsequent TKI therapy after PDC establishment (5 osimertinib, 3 first-generation EGFR-TKI, 2 entrectinib). Twelve PDCs established from these patients were screened with TKI which the patients were treated with (Fig. 2A). Two out of five patients with EGFR-mutant NSCLC were positive for *EGFR* T790M mutation, a marker of sensitivity to osimertinib, and received clinical benefits from osimertinib therapy, achieving a partial response (PR) and relatively long progression-free survival (PFS)^3^. Two corresponding PDCs (YU-1090 and YU-1073) exhibited *in vitro* sensitivity to osimertinib. Three PDCs (YU-1093, YU-1152, and YU-1094) generated from patients who were treated with osimertinib and had progressive disease as a best response were resistant to the drug (Fig. 2B). Three patients who received first-generation EGFR-TKI treatment did not achieve a partial response and had short PFS (n = 3). Accordingly, 4 corresponding PDCs (YU-1088, YU-1099, YU-1095, and YU-1091) were not responsive to gefitinib (Fig. 2C). Two patients with *ROS1*-positive NSCLC received entrectinib. One patient experienced a partial response with PFS of 6.5 months and corresponding PDC (YU-1080) was sensitive to entrectinib (Supplementary Fig. 3B). The other patient displayed cardiac toxicity to entrectinib therapy [not evaluable according to RECIST (Response Evaluation Criteria In Solid Tumors)] and switched to crizotinib. PFS on crizotinib was 4.2 months, indicating intrinsic resistance to the therapy (Fig. 2D). YU-1082 and YU-1083 cells were established from the patient before the start of crizotinib therapy and were resistant to the drug *in vitro*. A similar pattern was observed for YU-1085 cells that were established from the patient after crizotinib therapy (Fig. 2D and E). Together, these data suggest that PDCs may reflect patient treatment response to TKI.

### PDCs can guide the selection of potentially effective therapy in oncogene-driven lung adenocarcinoma

*BRAF* mutations are found in 1–3% of lung adenocarcinoma^2^. The two main types of *BRAF* mutations, V600E and non-V600E, are associated with different clinicopathological features of lung adenocarcinoma and exhibit different therapeutic response to BRAF-targeted targeted agents^1,28^. While dabrafenib alone or in combination with trametinib demonstrated promising efficacy in BRAF V600E mutant NSCLC, appropriate treatment paradigms are still under investigation for non-V600E mutations^29,30^.

**Figure 3:**
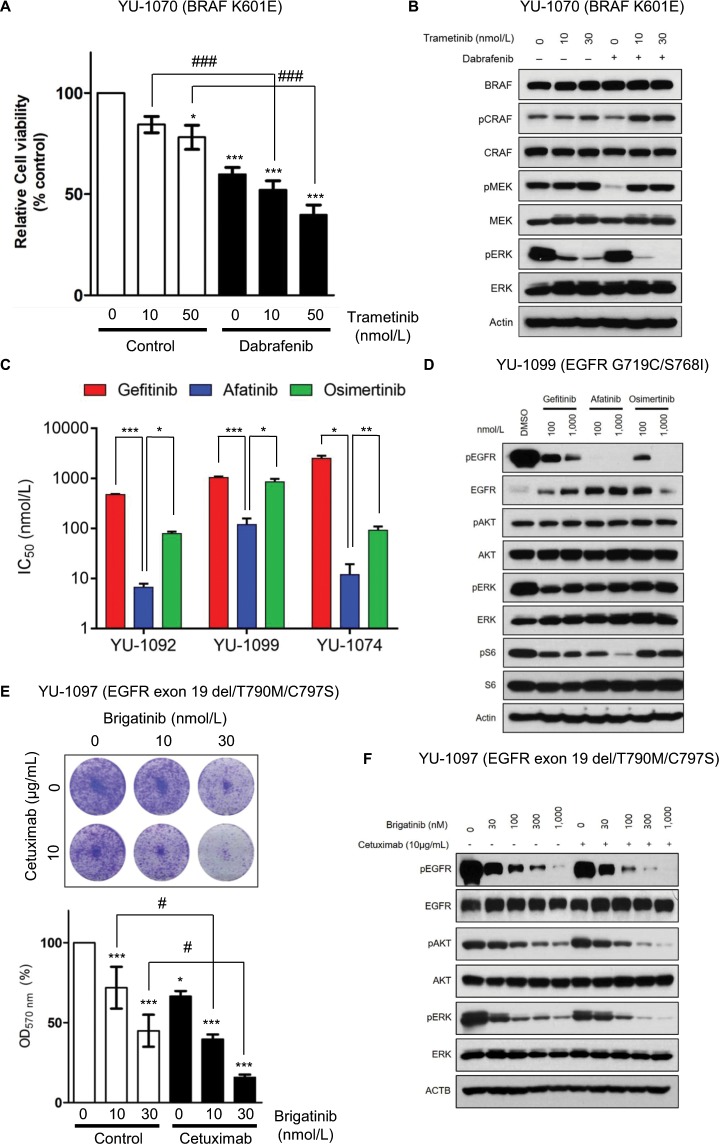
PDCs can guide the selection of potentially effective therapy in oncogene-driven lung adenocarcinoma. (**A**) YU-1070 cells were treated with the indicated concentrations of trametinib alone or in combination with dabrafenib. (ANOVA with Dunnett’s post test: *p < 0.05, ***p < 0.001 vs the value in DMSO control, ^###^p < 0.05 vs the value at the indicated comparison, n = 3) (**B**) YU-1070 cells were treated with the indicated concentrations of trametinib alone or in combination with dabrafenib for 1 hour. Cell lysates were immunoblotted with the indicated antibodies. (**C**) YU-1092, YU-1099, and YU-1074 cells were treated with the indicated concentrations of gefitinib, afatinib, or osimertinib. Data are presented as the mean ± SEM (n = 3) (two-tailed Student *t*-test: *p < 0.05, **p < 0.005 vs the value at the indicated comparison). n.s., not significant. (**D**) YU-1099 cells were treated with indicated concentrations of gefitinib, afatinib, or osimertinib for 24 hours. Cell lysates were immunoblotted with the indicated antibodies. (**E**) YU-1097 cells were treated with the indicated concentrations of brigatinib alone or in combination with cetuximab for 2 weeks. Colony formation was stained by crystal violet (upper panel). The bar graph shows quantification of the crystal violet staining (lower panel). (ANOVA with Dunnett’s post test: *p < 0.05, ***p < 0.001 vs the value in negative control, ^#^p < 0.05 vs the value at the indicated comparison, n = 3). (**F**) YU-1097 cells were treated with the indicated concentrations of brigatinib alone or in combination with cetuximab for 6 hours. Cell lysates were immunoblotted with the indicated antibodies. (**A** and **C**) Cell viability was measured by CellTiter-Glo. Data are presented as the mean ± SEM (n = 3). (**B**,** D**, and **F**) Immunoblots are representative of 3 independent experiments. The full-length blots can be found in Supplementary Fig. 6.

To identify an effective therapy for treatment of non-V600E *BRAF* mutant NSCLC, we tested efficacy of the single-agent and combination targeted therapy in YU-1070 cells harboring a *BRAF* K601E mutation. YU-1070 cells were highly resistant to vemurafenib, dabrafenib, and trametinib (Supplementary Fig. 5A). On the other hand, treatment with trametinib sensitized YU-1070 cells to dabrafenib (Fig. 3A). The combination of dabrafenib with trametinib induced c-Raf phosphorylation and completely blocked ERK phosphorylation (Fig. 3B). These data demonstrate that the *BRAF* K601E mutation may respond to the dabrafenib/trametinib combination therapy.

Most NSCLC patients harboring common *EGFR* mutations, such as deletions in exon 19 or the L858R mutation in exon 21, respond dramatically to EGFR-TKIs. However, there is a paucity of data regarding the activity of EGFR-TKIs in NSCLC harbor uncommon *EGFR* mutations, such as G719X, L861Q, S768I alone or in combination with each other, which occur in approximately 10% of *EGFR*-mutant NSCLC^31^.

To select the best treatment, we tested efficacy of EGFR-TKIs in YU-1092 cells (*EGFR* L861Q) and YU-1099 cells (*EGFR* G719C/S768I). Both YU-1092 and YU-1099 cells were resistant to gefitinib (Fig. 3C and D). Afatinib was the most potent drug with IC_50_ values of 6 nmol/L (*EGFR* L861Q) and 106 nmol/L (*EGFR* G719C/S768I) (Fig. 3C). Osimertinib was moderately effective against *EGFR* L861Q (IC_50_ = 75 nmol/L) and ineffective against the G719C/S768I complex mutation (IC_50_ = 836 nmol/L) (Fig. 3C). Afatinib more potently decreased EGFR and S6 phosphorylation compared with gefitinib and osimertinib in YU-1099 cells (Fig. 3D).

*EGFR* exon 20 insertions are among the rarer *EGFR* mutations (approximately 9% of *EGFR*-mutant NSCLC patients) and treatment for these mutations remain elusive without an approved inhibitor^32,33^. To identify optimal EGFR-TKIs, we investigated YU-1074 cells harboring the *EGFR* D770_N771insG mutation (Fig. 3C). Afatinib potently inhibited growth of YU-1074 cells, whereas osimertinib was less effective than afatinib (Fig. 3C). Together, these data suggest that afatinib among all EGFR-TKIs tested may be the most effective treatment for the uncommon *EGFR* mutations.

*EGFR* C797S mutation is one of the most commonly reported mechanisms of acquired resistance to third-generation EGFR-TKIs^5^. *EGFR* T790M mutation *in cis* to C797S mutation confers resistance to third-generation EGFR-TKIs as well as first-generation EGFR-TKIs^34^. A combination of brigatinib and cetuximab has been introduced to overcome the C797S-mediated resistance^35^. We aimed to evaluate EGFR-TKI efficacies in YU-1097 cells harboring an *EGFR* exon 19 del/T790M/C797S mutation (T790M *in cis* to C797S). YU-1097 cells were resistant to single-agent gefitinib, afatinib, osimertinib, and brigatinib (Supplementary Fig. 5B). Notably, YU-1097 cells were highly sensitive to the combination of brigatinib and cetuximab (Fig. 3E). The drug combination synergistically suppressed phosphorylation of AKT and ERK (Fig. 3F). These results show that the triple mutation may respond to the brigatinib/cetuximab combination therapy.

### AURKA as a potential therapeutic target in *EGFR*-mutant NSCLC resistant to third-generation EGFR-TKI

**Figure 4:**
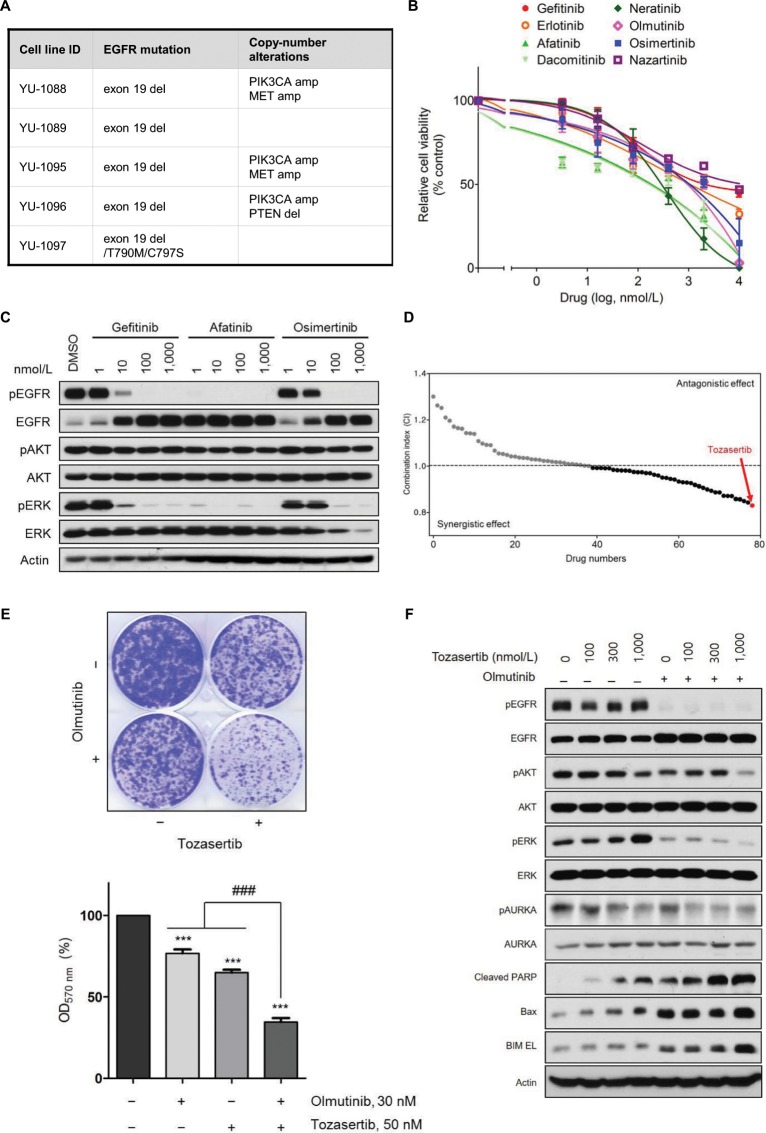
Aurora kinase A as a potential therapeutic target in EGFR-mutant NSCLC resistant to olmutinib. (**A**) Summary of WES analysis in PDCs resistant to third-generation EGFR-TKIs. Known mechanisms of resistance to osimertinib are shown. (**B**) Inhibition of YU-1089 cells by gefitinib (IC_50_ = 2,162 nmol/L), erlotinib (IC_50_ = 1,231 nmol/L), afatinib (IC_50_ = 275 nmol/L), dacomitinib (IC_50_ = 244 nmol/L), neratinib (IC_50_ = 275 nmol/L), olmutinib (IC_50_ = 857 nmol/L), osimertinib (IC_50_ = 1,013 nmol/L), and nazartinib (IC_50_ = 3,908 nmol/L). Cell viability was measured by CellTiter-Glo. Data are presented as the mean ± SEM (n = 3). (**C**) YU-1089 cells were treated with the indicated concentrations of gefitinib, afatinib, or osimertinib for 6 hours. Cell lysates were immunoblotted with the indicated antibodies. Immunoblots are representative of 3 independent experiments. (**D**) Combinatorial drug screening with 1 μM of olmutinib and 1 μM of each kinase inhibitor from the drug library was performed on YU-1089 cells to identify potent drug combinations. The x axis represents a number of kinase inhibitors used in this screen. The y axis represents combination index (CI) determined by the Bliss independence model. Each dot is the resulting CI for the individual drug. Gray dots indicate drugs with antagonism (CI > 1), whereas black dots indicate drugs with synergism (CI < 1). Tozasertib (red) exhibited the strongest synergistic effect. The screening identified 41 drugs with synergistic effects (CI < 1). (**E**) YU-1089 cells were treated with olmutinib alone, tozasertib alone, or a combination of olmutinib with tozasertib for 2 weeks. Colony formation was stained by crystal violet (upper panel). The bar graph (lower panel) shows quantification of the crystal violet staining. (ANOVA with Dunnett’s post test: ***p < 0.001 vs the value in negative control, ^###^p < 0.001 vs the value at the indicated comparison, n = 3). (**F**) YU-1089 cells were treated with the indicated concentrations of tozasertib or in combination with olmutinib for 24 hours. Cell lysates were immunoblotted with the indicated antibodies. Immunoblots are representative of 3 independent experiments. The full-length blots can be found in Supplementary Fig. 8.

Mechanisms of drug resistance remains elusive in 47–60% of patients with *EGFR*-mutant NSCLC progressing to third-generation EGFR-TKIs, posing a challenge to clinical decision making for these patients^36,37^. Using our clinically-relevant cell lines, we aimed to provide therapeutic strategies in this setting. In our panel of PDCs resistant to third-generation EGFR-TKIs, WES revealed genetic alterations (*EGFR* C797S, *MET* amplification, *PIK3CA* amplification, and *PTEN* loss) associated with osimertinib resistance^36–38^. However, known genetic alteration associated with drug response was not observed in YU-1089 cells (Fig. 4A, Supplementary Fig. 4A and E). First-, second-, and third-generation EGFR-TKIs failed to inhibit growth of YU-1089 cells (Fig. 4B). The EGFR-TKIs suppressed phosphorylation of EGFR and ERK but had no effect on phosphorylation of AKT (Fig. 4C).

To overcome the *EGFR*-independent mechanism of olmutinib resistance using YU-1089 cells, we comprised a panel of 79 investigational or FDA-approved drugs which target a wide range of kinases (Supplementary Table 5). Then, we performed drug combination screening on YU-1089 cells with olmutinib and each drug in the panel to nominate potent drug combinations. The screening identified 41 drugs with synergistic effects (CI < 1). The most strong synergy was observed with tozasertib, which targets Aurora kinases (Fig. 4D)^39^.

We next characterized the synergistic effect of combined *EGFR* and aurora kinase inhibition. The combination of olmutinib with tozasertib potently inhibited colony formation of YU-1089 cells compared to either agent alone (Fig. 4E). The robust synergism was confirmed in a 5 × 5 dose response matrix by using the Chou-Talalay method, resulting in a combination index (CI) value of 0.029 at 50% growth inhibition. Furthermore, the combination of olmutinib with tozasertib synergistically decreased phosphorylation of AKT and ERK and increased expression of apoptotic markers in YU-1089 cells. The comparable antitumor synergy was also shown by a combination of olmutinib with alisertib, a highly selective Aurora A kinase inhibitor under clinical development (CI = 0.196 at 44% growth inhibition), and a combination of osimertinib with tozasertib (CI = 0.189 at 52% growth inhibition) (Supplementary Fig. 7A)^40^. Recently, Shah *et al*. has shown that AURKA confers resistance to third-generation EGFR-TKIs in NSCLC and inhibition of AURKA can resensitize the tumor to EGFR-TKIs^41^. Thus, we tested if this drug combination strategy is applicable to other osimertinib-resistant PDCs. However, the osimertinib/alisertib combination was less potent in YU-1095, YU-1096, and YU-1097 cells than in YU-1089 cells (Supplementary Fig. 7B). These differential responses to combined EGFR and AUKRA inhibition may be due to difference in AURKA expression^41,42^. Supporting this hypothesis, AURKA expression was lower in PDCs that were not responsive to the drug combination (Supplementary Fig. 7C)^41,42^. Together, these results suggest that Aurora kinase A may be an actionable therapeutic target to overcome acquired resistance to third-generation EGFR-TKIs in *EGFR*-mutant NSCLC.

## Discussion

In this study, we established 23 PDCs that represent molecularly heterogeneous subsets of advanced lung adenocarcinoma. Among them, cell lines of *ROS1* fusions with various fusion partners, uncommon *EGFR* mutations, a resistant C797S mutation, and a rare *BRAF* mutation were included (Table 2)^5,43^. To the best of our knowledge, there are no commercially-available NSCLC cell lines endogenously harboring these mutations. Using novel cell lines, we presented effective therapeutic strategies which may inform future clinical decision making.

Selection of appropriate tumor specimens is important for successfully establishing patient-derived models^23,24^. Previous studies have shown that tumor cellularity in malignant effusions of NSCLC is highly variable, ranging from 0.1% to 90%^44^. Furthermore, cytological diagnosis of malignant effusions can be misleading because of potential mimics such as reactive mesothelial cells^45^. To use the malignant effusion as starting material, there is an urgent need to optimize establishment procedures. Our findings provided the evidence that both positive cytological diagnosis of malignancy (M+) and tumor colony formation (TCF+) were crucial to establishing PDCs from malignant effusions. Indeed, using M+/TCF+ malignant effusions can increase the success rate of PDC by approximately 2-fold (24.0% vs 48.8%). Additionally, M-/TCF−, M−/TCF+, and M+/TCF− malignant effusions (55/96, 57.3%) that have a low potential for establishing PDCs (3/55; 5.5%) can be excluded in a stepwise manner, thereby substantially reducing time and effort needed for sample processing and subsequent long-term culture. Carter *et al*. has shown that tumor cellularity in malignant effusions of advanced NSCLC is not correlated to sample volume^44^. Accordingly, we observed sample volume did not impact cytological diagnosis (*P* = 0.42372) or PDC establishment (*P* = 0.58232) (Table 1 and Supplementary Fig. 9). Although the difference was not statistically significant (OR = 0.5904, *P* = 0.3064) (Table 1), we observed a higher success rate in *EGFR* wild-type cases (31.0%) than *EGFR* mutant cases (20.9%). Similarly, John *et al*. and our group reported the negative correlation between EGFR mutations and NSCLC PDX model establishment from surgical resection, which may reflect a favorable prognostic value of EGFR mutations^46–48^. Interestingly, we noted tumor cell senescence in some M+/TCF+ primary cultures (11/41; 26.8%) between 4 to 7 passages. Despite high tumor purity, 5 PDCs became senescent between 10 to 23 passages, whereas other PDCs stably propagated over serial passage (Supplementary Table 3). These results show that some advanced lung adenocarcinoma (16/41; 39.0%) may depend on niche factors, which are not provided by R10 medium or autocrine signaling, for optimal growth. Notedly, recent study has utilized Wnt, FGF7, and FGF10 to establish NSCLC organoid models^49^. The success rate for organoids was higher than the success rate for PDX or PDC, implying that these specific factors may be associated with niche factor dependency observed in the subset of advanced lung adenocarcinoma^48,49^. Direct comparison between these patient-derived models may provide insight into tumorigenesis of NSCLC and therapeutic potential for targeting these niche factors and related signaling pathways.

To demonstrate clinical relevance, we tested efficacy of single-agent or combination targeted therapies in our PDCs harboring a *BRAF* K601E mutation and uncommon *EGFR* mutations (L861Q, G719C/S768I, D770_N771GinsG). Our data suggest that the *BRAF K601E* mutation may respond to a combination of dabrafenib and trametinib in a similar manner to a *BRAF V600E* mutation^29^. Indeed, the drug combination has demonstrated efficacy in a PDX model of *BRAF* K601E mutated melanoma^50^. Generally, NSCLC with uncommon *EGFR* mutation has been known to be less sensitive to first-generation EGFR-TKIs^31,51,52^. Similar to our findings in a PDC harboring the L861Q mutation, afatinib has shown lower IC_50_ values than first- or third-generation EGFR-TKIs in genetically-engineered Ba/F3 cells^31,53,54^. To our knowledge, we first reported *in vitro* efficacy of EGFR-TKIs against the *EGFR* G719C/S768I mutation and demonstrated that afatinib was the most potent among other EGFR-TKIs against the *EGFR* G719C/S768I mutation. These previous findings and ours corroborate the clinical activity of afatinib in patients with the uncommon mutations with an overall response rate (ORR) of 71.1% and median PFS of 10.7 months^32^.

However, we noted that IC_50_ values of osimertinib in YU-1092 cells and YU-1074 cells were comparable to the reported mean plasma concentration in patients receiving osimertinib (≈120 nmol/L), suggesting a potential of osimertinib against these mutations^55^. Consistent with our preclinical findings, osimertinib was shown to achieve an ORR of 60% in 5 patients with NSCLC harboring uncommon *EGFR* mutations (G719X, G719X/S768I, and L861Q)^56^. Additionally, nazartinib, a third-generation EGFR-TKI, also demonstrated preclinical activity against major variants of *EGFR* exon 20 insertions (D770_N771insSVD, V769_D770insASV, and H773_V774insNPH)^57^. More recently, a patient with lung adenocarcinoma harboring *EGFR* exon 20 insertion (S768_D770dup) was shown to respond to osimertinib^58^. Together, patients with NSCLC harboring an *EGFR* L861Q or D770_N771insG mutation may respond to osimertinib.

To date, heterogeneous mechanisms of osimertinib resistance have been reported^5^. Our data suggest that *EGFR* C797S-mediated resistance can be overcome by a combination of brigatinib and cetuximab, consistent with the previous finding^35^. Recent study has shown that overexpression of AURKA and its upstream TPX2 confers resistance to osimertinib and rociletinib^41^. Indeed, we found that combined inhibition of EGFR and AURKA is efficacious in YU-1089 cells that were established from patient tumor progressing to olmutinib (Fig. 4). It is plausible that YU-1089 cells responded to tozasertib and alisertib due to elevated expression of AURKA^41,42^.

We observed that EGFR-TKI treatment in EGFR-mutant PDCs increases total EGFR protein (Fig. 3D,F and 4C). Previous studies and ours imply that this phenomenon may be common among EGFR-TKI resistant cell lines, although a molecular mechanism behind the phenomenon remains unclear^34,35^. It is well established that inhibition of receptor tyrosine kinase (RTK) signaling pathway causes temporary relief of RTK-dependent negative feedback mechanisms, resulting in a rebound in RTK expression or downstream signaling activation^59,60^. EGFR signaling is regulated by various EGFR inducible negative regulators such as LRIG1, MIG6, SOCS4, and SOC5^61^. Furthermore, LRIG1 and MIG6 are overexpressed in EGFR-mutant NSCLC cell lines and function as a negative regulator of EGFR signaling^61–63^. These findings may suggest a possible involvement of EGFR inducible negative regulators in EGFR upregulation after EGFR-TKI treatment. Further studies are required to investigate mechanisms of the EGFR rebound and its relationship to EGFR-TKI resistance^59,60^.

This study had several limitations. Previous studies have demonstrated that long-term culture of patient-derived models results in accumulation of somatic mutations and subclonal selection. Occasionally, these genetic drifts may functionally impact drug sensitivity^26,27^. PDCs in our study varied in their growth rates and time they take to achieve high tumor purity (Supplementary Table 3). We observed that majority of PDCs at early passages (1 to 8, median passage number of 3) were contaminated with fibroblasts (0%–51.9%, median value of 3.94%) in line with previous findings (Supplementary Table 1)^8^. Because of differential trypsinization, fibroblasts did not overgrow tumor cells, although fibroblast contamination generally resulted in additional cell passaging and a delay in functional tests (Supplementary Tables 1 and 3). Particularly, drug testing in 1 PDC was only available after 20 passages, which may not well represent patient tumor. Therefore, improved culture conditions should be tested in M+/TCF+ malignant effusions to accelerate tumor growth and turn-around time for functional assays. We also acknowledge that the presented therapeutic strategies should be validated in prospective clinical studies.

In summary, we streamlined a protocol for establishing PDCs and showed that these PDCs can be valuable preclinical platforms for designing therapeutic strategies.

## Supplementary information

Supplementary Figures and Tables

## Data Availability

Materials and data are available upon reasonable request to corresponding authors.
