# Supplementary material for: Patient-Derived Cells to Guide Targeted Therapy for Advanced Lung Adenocarcinoma
Source: Sci Rep. 2019 Dec 27;9:19909. doi: 10.1038/s41598-019-56356-4 (PMC6934824; doi:10.1038/s41598-019-56356-4)
Supplement: Supplementary file 1 — Supplementary Figures and Tables [file 41598_2019_56356_MOESM1_ESM.pdf]

Patient-Derived Cells to Guide Targeted Therapy for Advanced Lung Adenocarcinoma

Seok-Young Kim[a,b], Ji Yeon Lee[b], Dong Hwi Kim[a,b], Hyeong-Seok Joo[a,b], Mi Ran Yun[a,b], Dongmin Jung[c], Jiyeon Yun[b], Seong Gu Heo[b], Beung-Chul Ahn[b], Chae Won Park[b], Kyoung Ho Pyo[b], You Jin Chun[b], Min Hee Hong[b], Hye Ryun Kim[b*], Byoung Chul Cho[b*]

[a]*JEUK Institute for Cancer Research, JEUK Co.,Ltd., Gumi-City, Kyungbuk, Korea*
[b]*Yonsei Cancer Center, Division of Medical Oncology, Department of Internal Medicine, Yonsei University College of Medicine, Seoul, Korea*
[c]*Severance Biomedical Science Institute, Yonsei University of College of Medicine, Seoul, Korea*

[*]**Corresponding author:** Byoung Chul Cho, MD, PhD, Yonsei Cancer Center, Division of Medical Oncology, Department of Internal Medicine, Yonsei University College of Medicine, 50 Yonsei-Ro, Seodaemun-Gu, Seoul, Korea. Phone: 82-2-2228-0870; Fax: 82-2-393-3562; E-mail: CBC1971@yuhs.ac. Hye Ryun Kim, MD, Yonsei Cancer Center, Division of Medical Oncology, Department of Internal Medicine, Yonsei University College of Medicine, 50 Yonsei-Ro, Seodaemun-Gu, Seoul, Korea. Phone: 82-2-2228-0870; Fax: 82-2-393-3562; E-mail: NOBELG@yuhs.ac

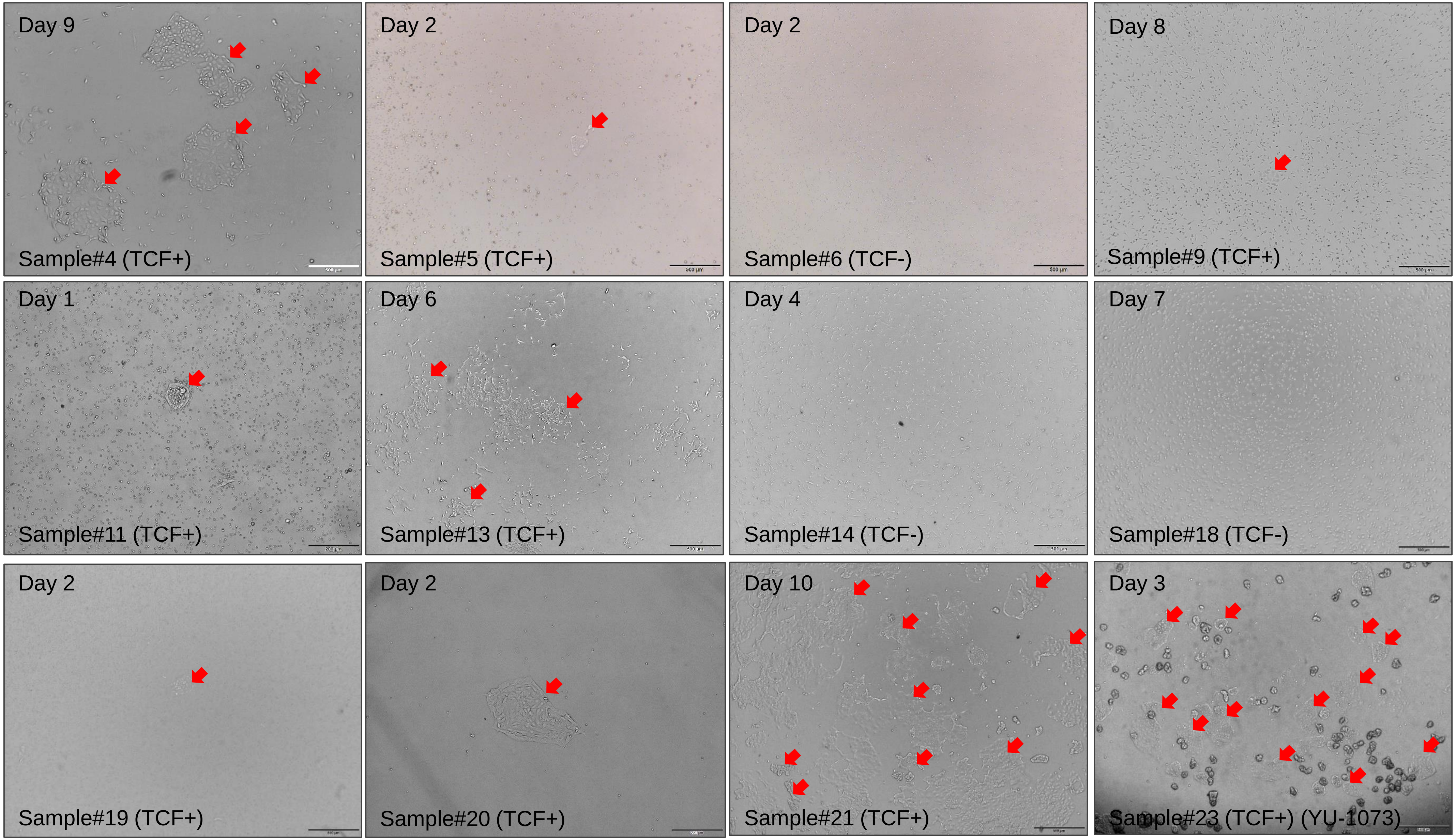

Supplementary Figure 1. Adherent tumor cells with an epithelial cell morphology, or tumor colony, were occasionally observed in primary cultures within 1 to 10 day s of culture initiation. Light microscopic pictures showing 12 representative cases of primary cultures. A number of days after culture initiation is described for each case. Tumor colonies are indicated as red arrows.

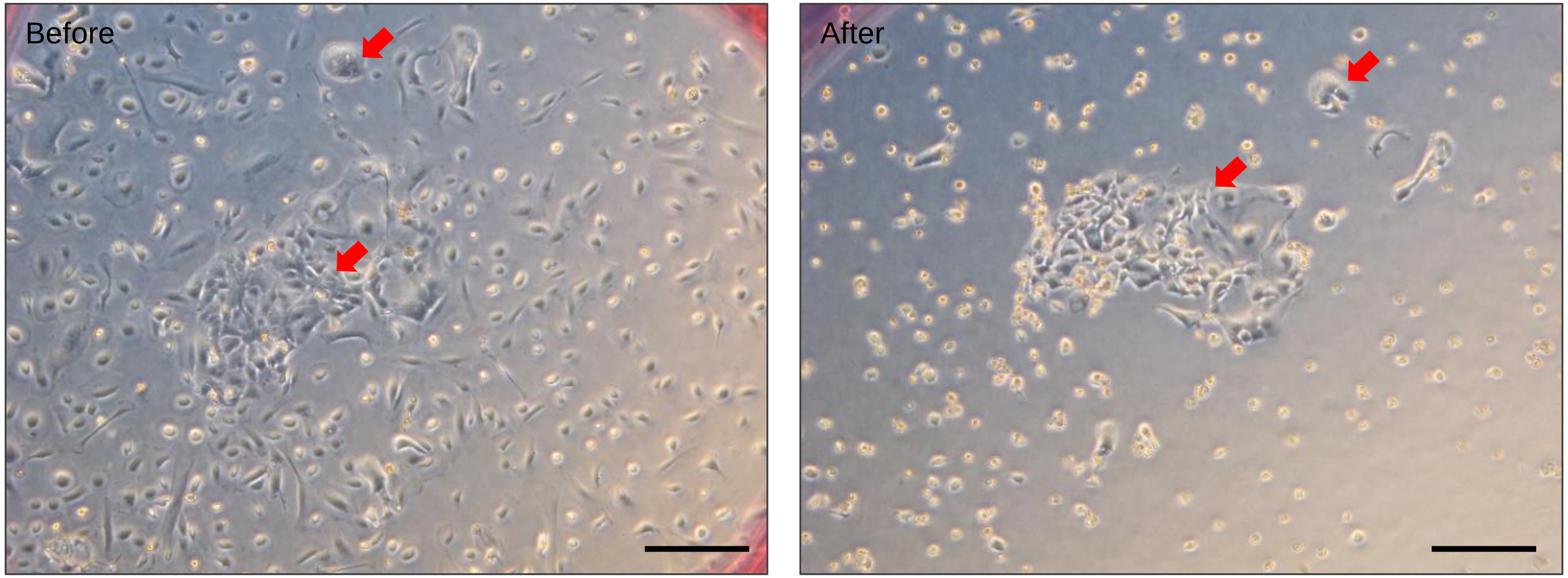

Supplementary Figure 2. Differential trypsinization method to eradicate the contamination. Representative light microscopic data showing a primary culture (sample#83) contaminated with stromal cells (upper) (scale bar, 500 µm). Tumor colonies are indicated as red arrows. Differential trypsinization was applied to the primary culture to remove the stromal cells (lower). In general, a primary culture was washed with PBS and treated with 0.25% Trypsin-EDTA (Thermo Fisher Scientific, Waltham, MA) that was diluted with PBS at a ratio of 1:2 to 1:10. Then, the primary culture was incubated for 0.5 to 10 minutes at room temperature to a time point when tumor colonies remain attached to and stromal cells detached from a cell culture plate. Next, supernatant containing stromal cells were discarded. The cell culture plate containing tumor colonies were carefully washed with R10 medium and replenished with fresh R10 medium.

A

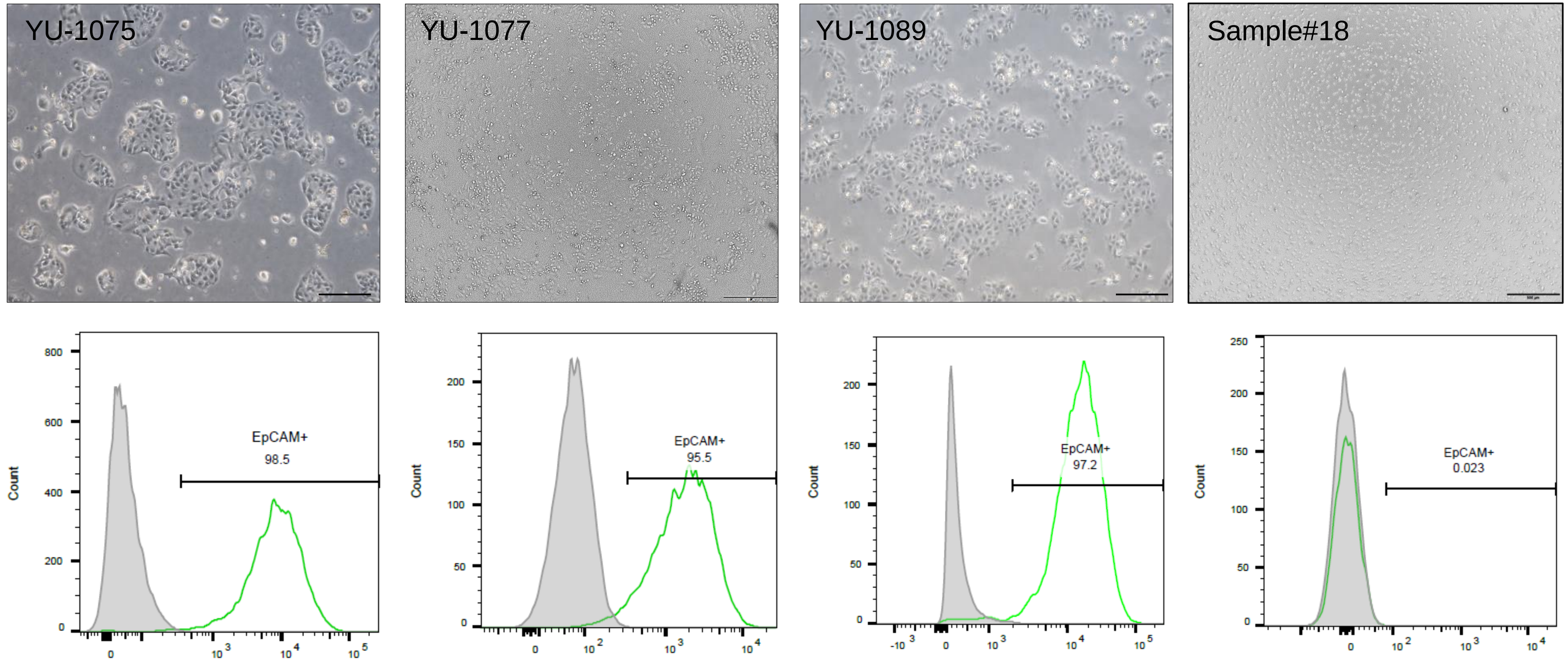

B

Cell line ID	*In vitro* sensitivity to entrectinib (IC_{50}, nmol/L)	Prior TKI therapy	Subsequent TKI therapy	Best response by RECIST criteria	PFS on subsequent therapy (Mo)	Response in the clinic vs *in vitro* sensitivity
YU-1080	5	N/A	Entrectinib	PR	6.54	Matching

Supplementary Figure 3. Establishment of PDCs. (A) Light microscopic pictures showing representative cases of successful (YU-1075, YU-1077, and YU-1089) or failed (Sample#18) primary cultures (scale bar, 500 μm). Successful primary cultures were free of stromal cells and contained tumor cells compared to the failed primary culture (upper panel). Primary cultures were stained with matching isotype control (grey curve) or PE-conjugated human EpCAM antibody (green curve) and analyzed by flow cytometry (lower p anel). The x-axis shows fluorescence intensity and the y-axis shows the number of stained cells. A proportion of EpCAM-expressing cells were >95% in successful cases, wherea s the proportion was extremely scarce in the sample#18. (B) Table showing *in vitro* sensitivity to entrectinib in YU-1080 cells and clinical outcome in the corresponding patient. N/A , not available; PR, partial response; PFS, progression-free survival.

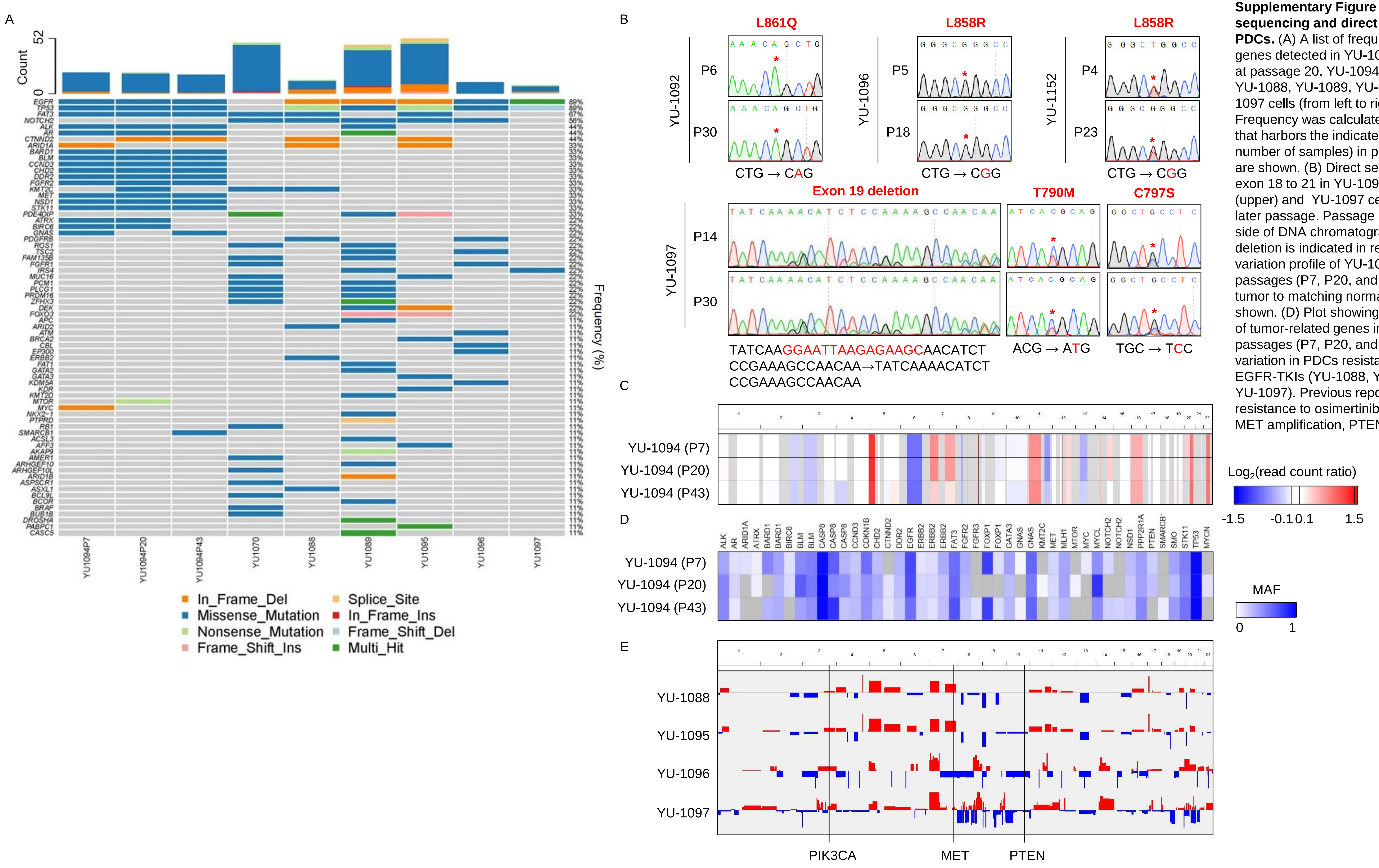

Supplementary Figure 4. Whole-exome sequencing and direct sequencing analysis in PDCs. (A) A list of frequently mutated tumor-related genes detected in YU-1094 at passage 7, YU-1094 at passage 20, YU-1094 at passage 43, YU-1070, YU-1088, YU-1089, YU-1095, YU-1096, and YU-1097 cells (from left to right) (see methods). Frequency was calculated as (a number of sample that harbors the indicated genetic alteration)/(a total number of samples) in percent value. Top 70 genes are shown. (B) Direct sequencing analysis of EGFR exon 18 to 21 in YU-1092, YU-1096, YU-1152, (upper) and YU-1097 cells (lower) at early and later passage. Passage number is indicated on left side of DNA chromatogram. Base change or exon deletion is indicated in red. (C) Copy number variation profile of YU-1094 cells at multiple passages (P7, P20, and P43). Read count ratio of tumor to matching normal blood on the log2 scale is shown. (D) Plot showing mutation allele frequency of tumor-related genes in YU-1094 cells at multiple passages (P7, P20, and P43). (E) Copy number variation in PDCs resistant to third generation EGFR-TKIs (YU-1088, YU-1095, YU-1096, and YU-1097). Previous reported mechanisms of resistance to osimertinib (PIK3CA amplification, MET amplification, PTEN loss) are indicated.

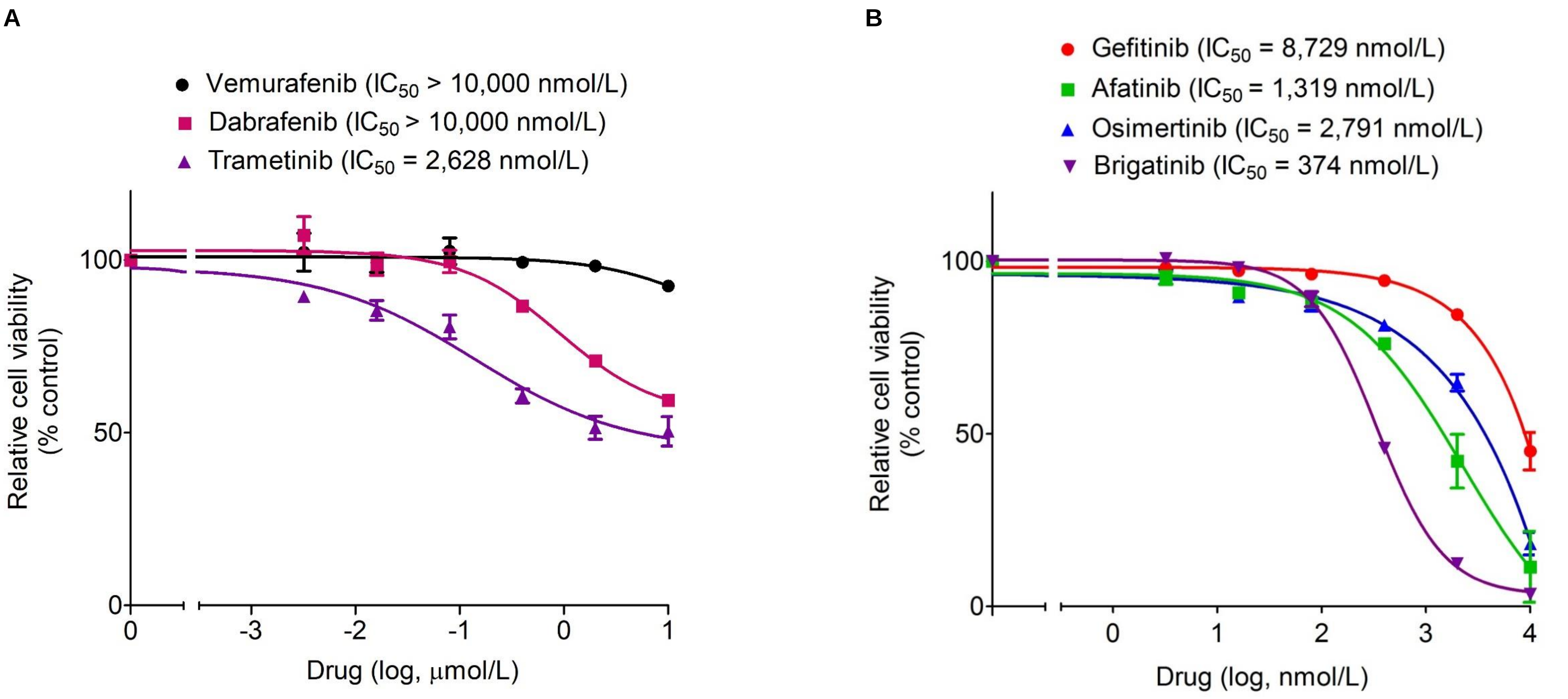

Supplementary Figure 5. ***In vitro* response to TKIs in YU-1070 cells and YU-1097 cells.** (A) YU-1070 cells were treated with the indicated concentrations of vemurafenib, darafenib, or trametinib alone. Cell viability was measured by CellTiter-Glo. Data are presented as the mean ± SEM (n = 3). (B) YU-1097 cells were treated with the indicated concentrations of gefitinib, afatinib, osimertinib, or brigatinib. Cell viability was measured by CellTiter-Glo. Data are presented as the mean ± SEM (n = 3).

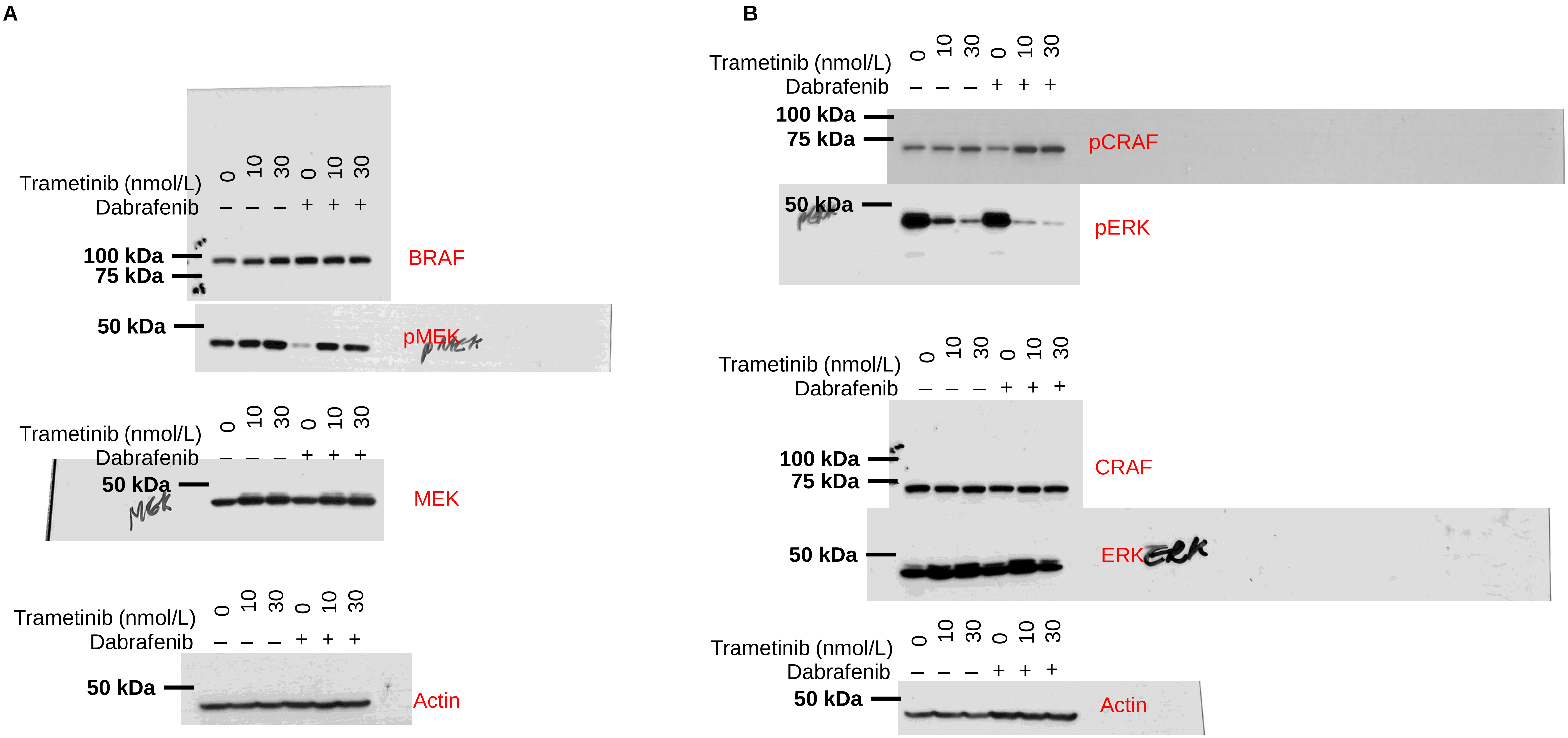
A
Trametinib (nmol/L) 0 10 30 0 10 30
Dabrafenib – – – + + +
100 kDa
75 kDa
BRAF
50 kDa
pMEK
Trametinib (nmol/L) 0 10 30 0 10 30
Dabrafenib – – – + + +
50 kDa
MEK
Trametinib (nmol/L) 0 10 30 0 10 30
Dabrafenib – – – + + +
50 kDa
Actin
B
Trametinib (nmol/L) 0 10 30 0 10 30
Dabrafenib – – – + + +
100 kDa
75 kDa
pCRAF
50 kDa
pERK
Trametinib (nmol/L) 0 10 30 0 10 30
Dabrafenib – – – + + +
100 kDa
75 kDa
CRAF
50 kDa
ERK
Trametinib (nmol/L) 0 10 30 0 10 30
Dabrafenib – – – + + +
50 kDa
Actin

C

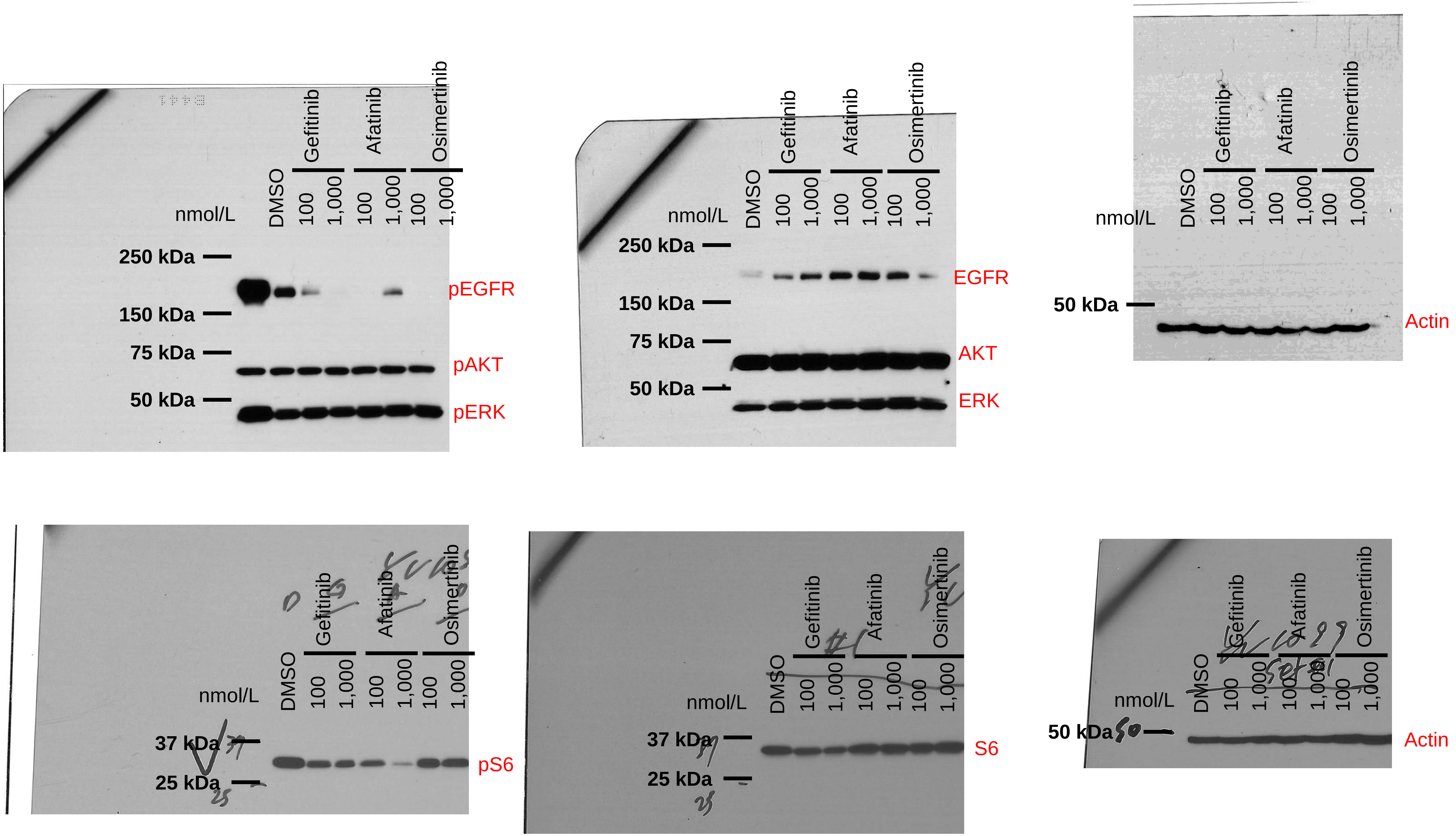

Gefitinib
Afatinib
Osimertinib
nmol/L
DMSO
100
1,000
100
1,000
100
1,000
250 kDa
150 kDa
75 kDa
50 kDa
pEGFR
pAKT
pERK
EGFR
AKT
ERK
Actin
37 kDa
25 kDa
pS6
S6

D

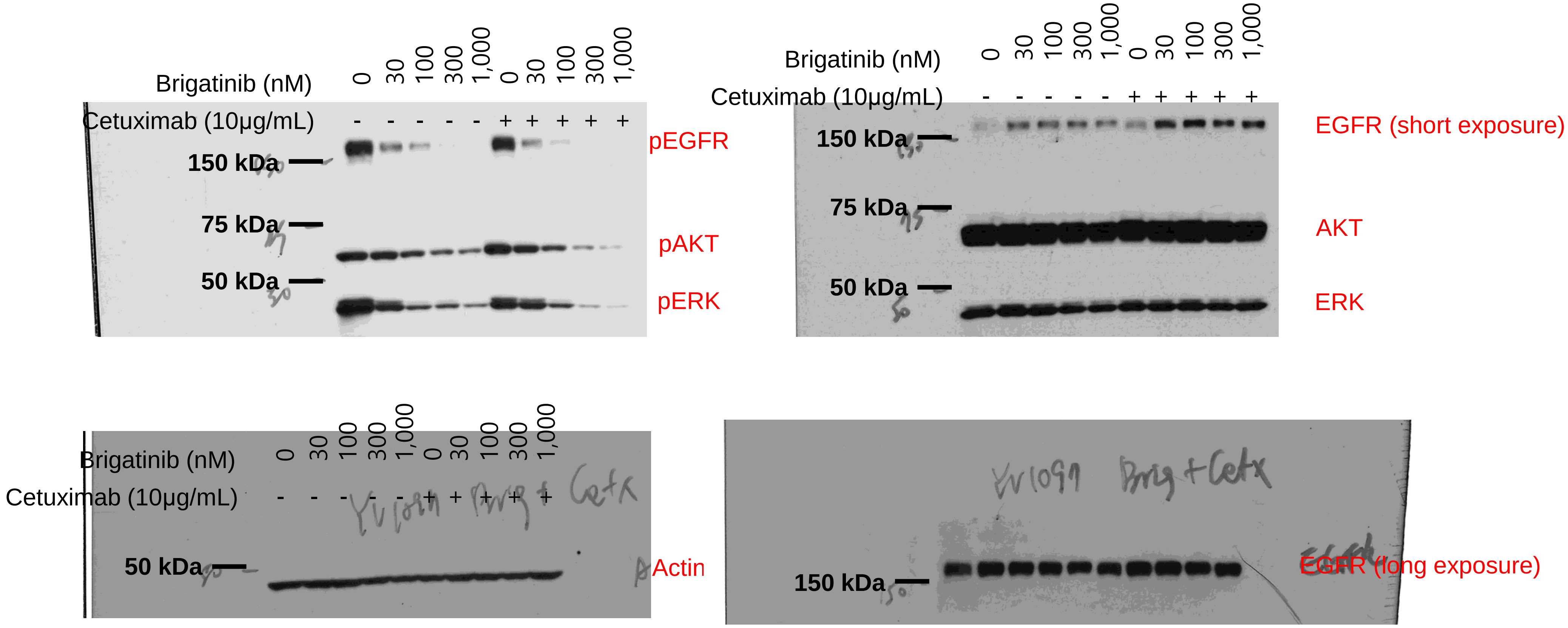

Supplementary Figure 6. Full-length blots related to Figure 3. (A) Immunoblots to assess BRAF and phospho-MEK in YU-1070 cells. Membranes were stripped and re-blotted for MEK and Actin. (B) Immunoblots to assess phospho-CRAF and phospho-ERK in YU-1070 cells. Membranes were stripped and re-blotted for total CRAF, total ERK, and Actin. (C) Immunoblots to assess phospho-EGFR, phospho-AKT, phospho-ERK, and phosphor-S6 in YU-1099 cells. Membranes were stripped and re-blotted for total EGFR, total AKT, total ERK, total S6 and Actin. (D) Immunoblots to assess phospho-EGFR, phospho-AKT, and phospho-ERK in YU-1097 cells. Membranes were stripped and re-blotted for total EGFR, total AKT, total ERK, and Actin.

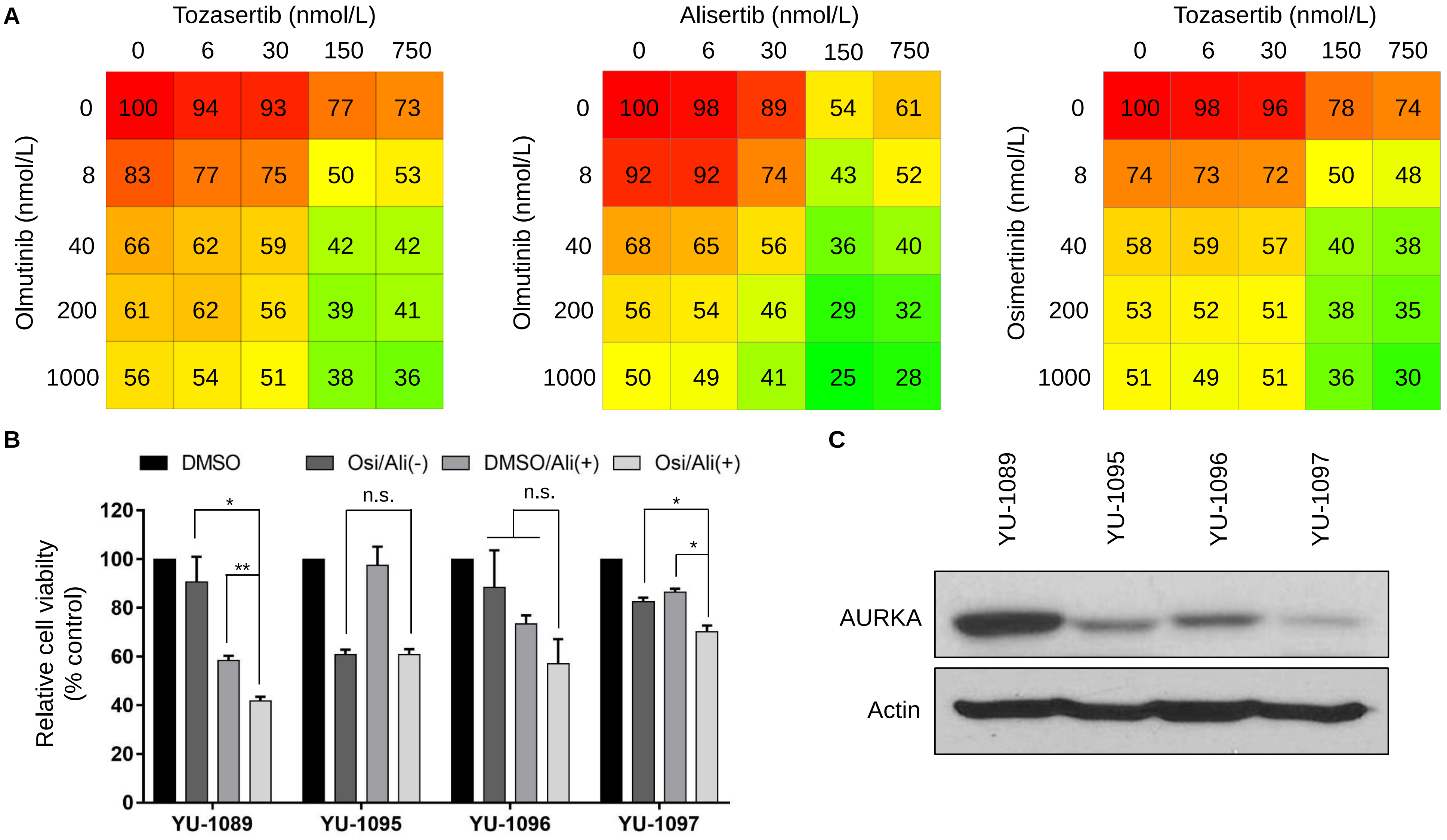

Supplementary Figure 7. Combined EGFR and AURKA inhibition overcomes resistance to third-generation EGFR-TKIs in YU-1089 cells. (A) 5 x 5 dose response matrices showing responses of YU-1089 cells to a combination of olmutinib and tozasertib (left), olmutinib and alisertib (middle), and osimertinib and tozasertib (right) at the indicated concentrations of the drugs. Response values are relative cell viability compared to DMSO control. Cell viability was measured by CellTiter-Glo. Data are presented as the mean (n = 3). Combination index (CI) was calculated using the Chou-Talalay method. (B) YU-1089, YU-1095, YU-1096, and YU-1097 cells were treated with osimertinib at 200 nM with or without alisertib (150 nM). Cell viability was measured by CellTiter-Glo. Data are presented as the mean ± SEM (n = 3) (two-tailed Student *t*-test: *p<0.05, **p<0.005 vs the value at the indicated comparison). n.s., not significant. (C) Endogenous expression of AURKA in YU-1089, YU-1095, YU-1096, and YU-1097 cells. Cell lysates were immunoblotted with the indicated antibodies. Full-length blots can be found in Supplementary Figure 8.

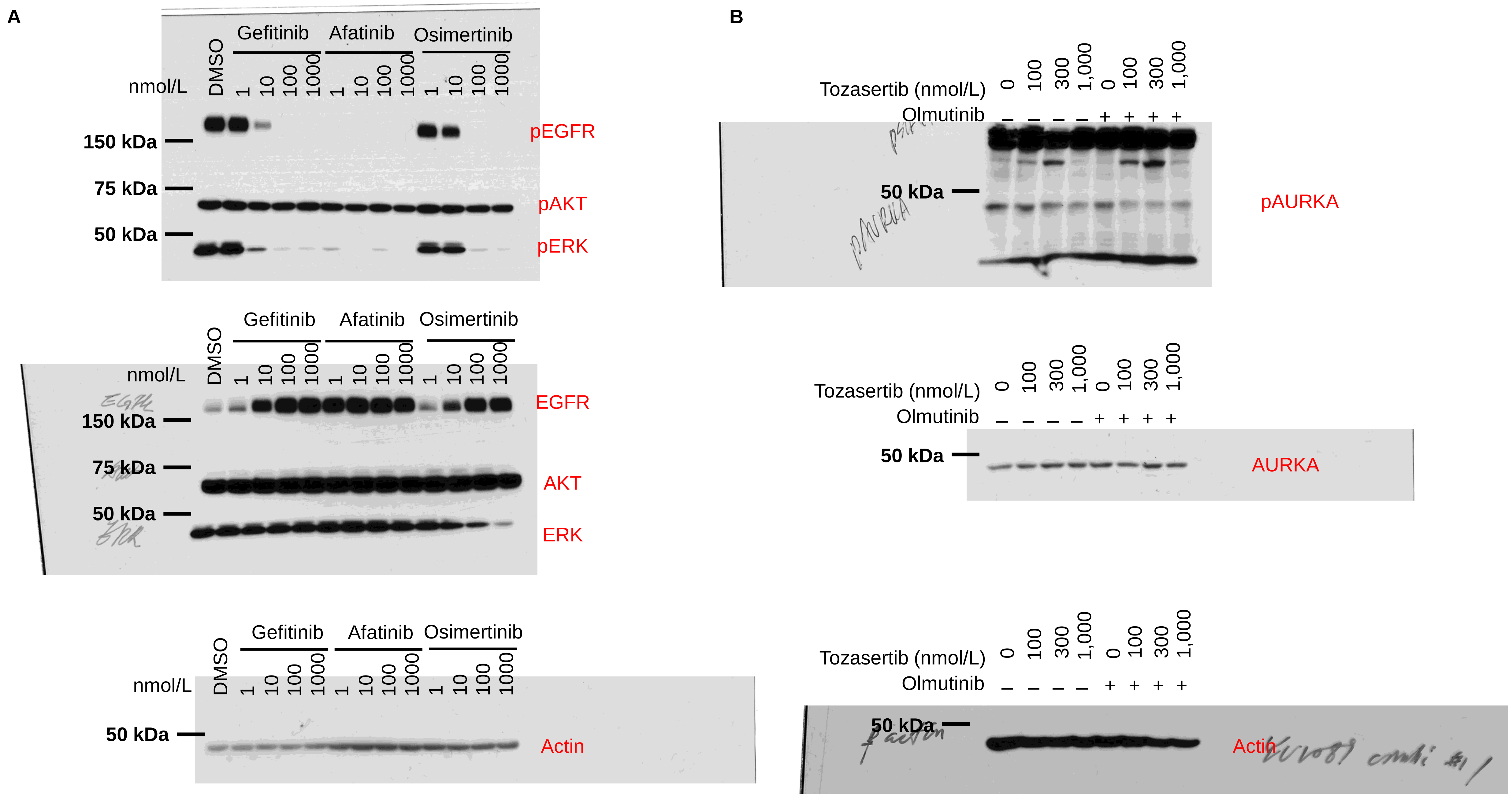
A
Gefitinib
Afatinib
Osimertinib
DMSO
nmol/L
1
10
100
1000
150 kDa
75 kDa
50 kDa
pEGFR
pAKT
pERK
EGFR
AKT
ERK
Actin
B
Tozasertib (nmol/L)
0
100
300
1,000
Olmutinib
pAURKA
AURKA
Actin

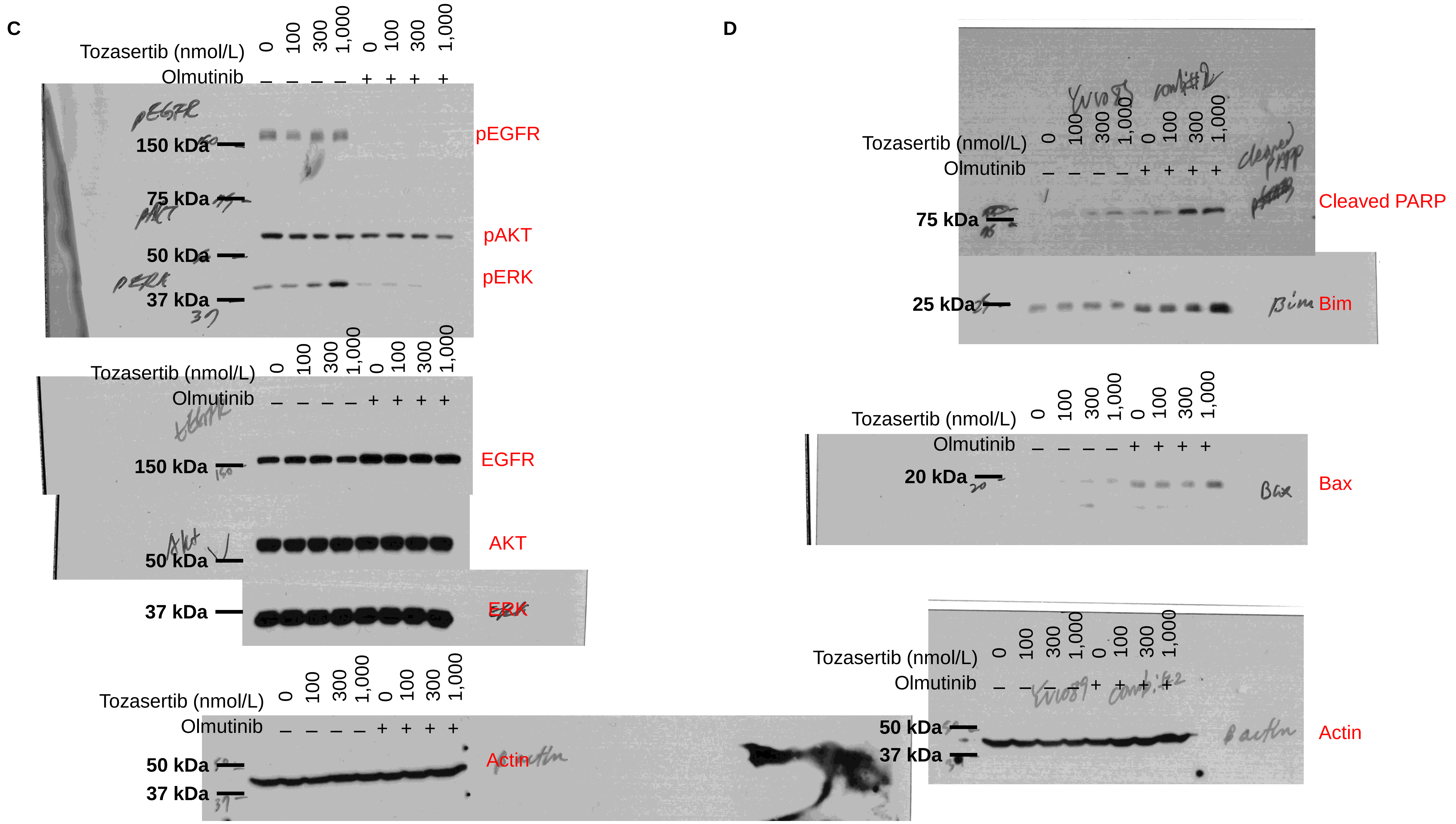
C
Tozasertib (nmol/L) 0 100 300 1,000 0 100 300 1,000
Olmutinib – – – – + + + +
150 kDa
75 kDa
50 kDa
37 kDa
pEGFR
pAKT
pERK
Tozasertib (nmol/L) 0 100 300 1,000 0 100 300 1,000
Olmutinib – – – – + + + +
150 kDa
50 kDa
37 kDa
EGFR
AKT
ERK
Tozasertib (nmol/L) 0 100 300 1,000 0 100 300 1,000
Olmutinib – – – – + + + +
50 kDa
37 kDa
Actin
D
Tozasertib (nmol/L) 0 100 300 1,000 0 100 300 1,000
Olmutinib – – – – + + + +
75 kDa
25 kDa
Cleaved PARP
Bim
Tozasertib (nmol/L) 0 100 300 1,000 0 100 300 1,000
Olmutinib – – – – + + + +
20 kDa
Bax
Tozasertib (nmol/L) 0 100 300 1,000 0 100 300 1,000
Olmutinib – – – – + + + +
50 kDa
37 kDa
Actin

E

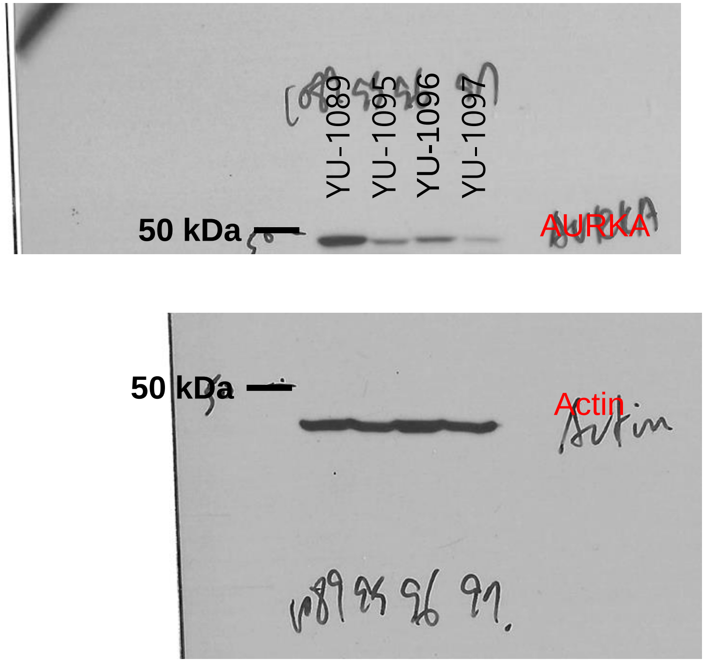

Supplementary Figure 8. Full-length blots related to Figure 4 and Supplementary Figure 7. (A) Immunoblots to assess phospho-EGFR, phospho-AKT, and phospho-ERK in YU-1089 cells. Membranes were stripped and re-blotted for total EGFR, total AKT, total ERK, and Actin. (B) Immunoblots to assess phospho-AURKA in YU-1897 cells. Membranes were stripped and re-blotted for total AURKA and Actin. (C) Immunoblots to assess phospho-EGFR, phospho-AKT, and phospho-ERK in YU-1089 cells. Membranes were stripped and re-blotted for total EGFR, total AKT, total ERK, and Actin. (D) Immunoblots to assess cleaved PARP and Bim in YU-1089 cells. Membranes were stripped and re-blotted for Bax and Actin. (E) Immunoblots to assess AURKA in YU-1089, YU-1095, YU-1096, and YU-1097 cells. Membranes were stripped and re-blotted for Actin.

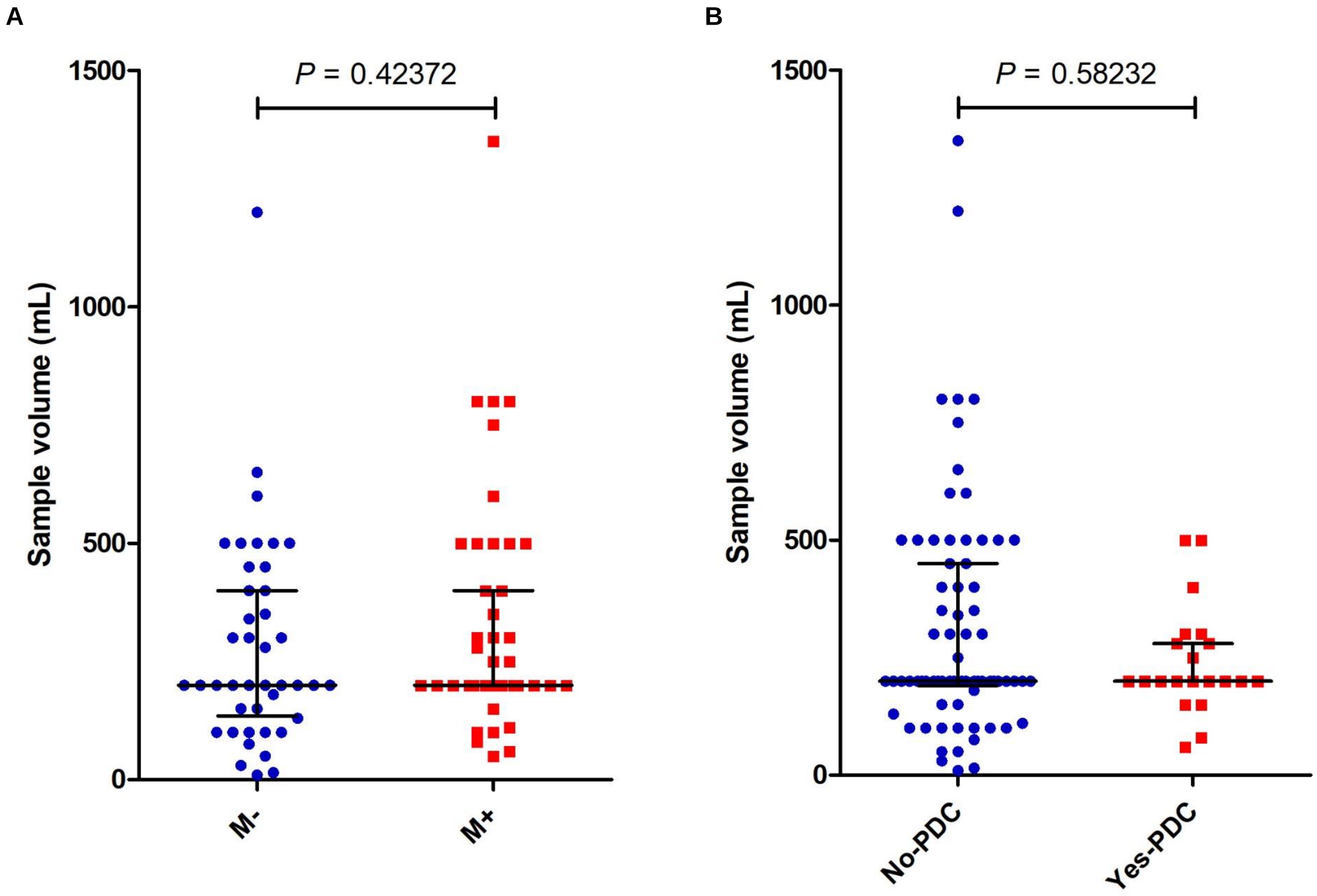

Supplementary Figure 9. Sample volume of malignant effusions was not correlated with cytological diagnosis or PDC establishment. (A) A graph showing sample volume and cytological diagnosis classification across 96 malignant effusions. Each dot represents a malignant effusion. P value was calculated using Mann-Whitney U test. (B) A graph showing sample volume and PDC establishment across 96 malignant effusions. Each dot represents a malignant effusion. P value was calculated using Mann-Whitney U test.

Supplementary Table 1. Fibroblast contamination in PDCs at early passages.

Cell line ID	Cell line description	Passage number at which flow cytometry analysis was performed	Fibroblast contamination assessed by flow cytometry analysis (%)
YU-1073	Sample#23	3	9.36
YU-1074	Sample#34	8	0
YU-1090	Sample#49	1	9.27
YU-1094	Sample#55	2	1.08
YU-1099	Sample#94	3	1.17
YU-1152	Sample#96	5	0
YU-1091	Sample#66	6	3.94
YU-1088	Sample#39	1	39.7
YU-1095	Sample#59	5	2.14
YU-1096	Sample#83	4	51.9
YU-1075	Sample#27	1	5.8
YU-1077	Sample#7	4	44.3
YU-1080	Sample#33	1	2.11
YU-1081	Sample#52	1	31.3
YU-1082	Sample#62	2	1.93
YU-1083	Sample#68	1	14.8
YU-1085	Sample#90	4	0.88
HCC827	Human NSCLC cell line	N/A	0.3
H2291	Human NSCLC cell line	N/A	0.064
MRC-5	Human fibroblast cell line	N/A	98.8

Flow cytometry analysis was performed on primary cultures that grew fast and had sufficient number of cells.

N/A, not available.

Supplementary Table 2. List of primers used in this study.

Primer name	Direction	Primer sequence
H-EGFR-Ex18_M13	Forward	GTAAAACGACGGCCAGTAGGGCTGAGGTGACCCTTGT
H-EGFR-Ex18_M13	Reverse	GCGGATAACAATTTCACACAGGCCCCACCAGACCATGAGAG
H-EGFR-Ex19_M13	Forward	GTAAAACGACGGCCAGTACCATCTCACAATTGCCAGTTAAC
H-EGFR-Ex19_M13	Reverse	GCGGATAACAATTTCACACAGGGAGGTTCAGAGCCATGGACC
H-EGFR-Ex20_M13	Forward	GTAAAACGACGGCCAGTCATGTGCCCCTCCTTCTGG
H-EGFR-Ex20_M13	Reverse	GCGGATAACAATTTCACACAGGTATCTCCCCTCCCCGTATCTC
H-EGFR-Ex21_M13	Forward	GTAAAACGACGGCCAGTGAATTCGGATGCAGAGCTTCTTC
H-EGFR-Ex21_M13	Reverse	GCGGATAACAATTTCACACAGGATGCTGGCTGACCTAAAGCC
H-ALK-F	Forward	CCT GAG TAC AAG CTG AGC AAG CT
H-ALK-R1	Reverse	CAAATACTGACAGCCACAGGCAA
H-ALK-R2	Reverse	CGGTGTTGATTACATCCGGG
H-ALK-R3	Reverse	TGATTACATCCGGGTCCTGG
H-ALK-R4	Reverse	ACAAGTGGACCATATTCTATCGGC
H-SLC34A2-kinase-F	Forward	GGATTGGGAGATTGATTTTACTTCTC
H-SLC34A2-kinase-R	Reverse	TTCTGAATAACTGAAGTTGGTCCTG
H-TPM3-280F-62-1631	Forward	GGT GGC CTC CTT GAA CCG TA
H-TPM3-R-60-1631	Reverse	TCA CAT CGC CAT CTT CAC CT
H-CD74-409F-60-1789	Forward	GGC AAC ATG ACA GAG GAC CA
H-CD74-R-60-1789	Reverse	ACC CTT CTC GGT TCT TCG TT

Supplementary Table 3. Cell passaging in PDCs.

Cell line ID	Passage numbers used in testing after achieving high tumor purity	Maximum passage number achieved	Last passage number before culture was discontinued
YU-1070	12 - 18	22	22
YU-1073	6 - 12	N/D	27
YU-1074	8 - 10	15	15
YU-1090	12 - 17	N/D	23
YU-1092	16 - 28	N/D	28
YU-1093	17 - 19	N/D	19
YU-1094	17 - 32	N/D	40
YU-1099	12 - 38	N/D	40
YU-1152	5 - 25	N/D	35
YU-1091	7 - 11	11	11
YU-1088	33 - 34	N/D	36
YU-1089	13 - 26	N/D	51
YU-1095	16 - 25	N/D	37
YU-1096	6 - 19	N/D	20
YU-1097	14 - 33	N/D	34
YU-1075	3 - 10	10	10
YU-1076	10 - 28	N/D	35
YU-1077	11 - 23	N/D	35
YU-1080	1 - 13	13	13
YU-1081	12 - 20	N/D	24
YU-1082	10 - 24	N/D	44
YU-1083	15 - 33	N/D	33
YU-1085	12 - 24	N/D	31

N/D, not determined.

Supplementary Table 4. Clinical annotations and characteristics of established PDCs.

Cell line ID	Clinical annotations					PDC characteristics	
	Patient treatment response to TKIs			Known patient tumor mutation[a]		Driver mutation[d]	WES
	Prior TKI therapy	Best response to the prior TKI	PFS on the prior TKI (Months)	Driver mutation before disease progression to the prior TKI	Driver mutation after disease progression to the prior TKI		
YU-1070	N/A	N/A	N/A	N/A	N/A	BRAF K601E	Yes
YU-1073	Gefitinib	PR	7.0	N/A	EGFR L858R/T790M	EGFR L858R/T790M	No
YU-1074	Gefitinib	PD	1.0	EGFR exon 20 insertion	N/A	EGFR D770_N771insG	No
YU-1090[h]	Gefitinib	PR	18.2	EGFR L858R	EGFR L858R/T790M	EGFR L858R/T790M	No
YU-1092	Gefitinib	PD	1.9	EGFR L861Q	N/A	EGFR L861Q	No
YU-1093	Erlotinib	PD	1.8	EGFR exon 19 deletion	EGFR exon 19 deletion	EGFR exon 19 deletion	No
YU-1094	Gefitinib	PR	14.8	EGFR L858R	EGFR L858R	EGFR L858R	Yes
YU-1099	Gefitinib	PR	13.6	EGFR G719X/S768I	EGFR G719X/S768I	EGFR G719C/S768I	No
YU-1152	Erlotinib	PD	1.4	EGFR L858R	EGFR L858R	EGFR L858R	No
YU-1091	Afatinib + Ruxolitinib	SD	3.2	EGFR L858R	EGFR L858R	EGFR L858R	No
YU-1088[i]	Osimertinib	PR	21.3	EGFR exon 19 deletion	EGFR exon 19 deletion	EGFR exon 19 deletion	Yes
YU-1089	Olmutinib	PR	7.6	EGFR exon 19 deletion/T790M	EGFR exon 19 deletion	EGFR exon 19 deletion	Yes
YU-1095[i]	Osimertinib	PR	21.3	EGFR exon 19 deletion	EGFR exon 19 deletion	EGFR exon 19 deletion	Yes
YU-1096[h]	Osimertinib	PR	8.8	EGFR L858R/T790M	N/A	EGFR L858R	Yes
YU-1097	Osimertinib	PR	38.7	EGFR exon 19 deletion	N/A	EGFR exon 19 deletion/T790M/ C797S	Yes
YU-1075[j]	Crizotinib	PD	1.9	ALK fusion[b]	N/A	EML4-ALK	No
YU-1076[j]	Ceritinib	NE[e]	NE	ALK fusion[b]	N/A	EML4-ALK	No
YU-1077	Alectinib	PR	17.7	ALK fusion[b]	N/A	EML4-ALK G1202R	No
YU-1080	N/A	N/A	N/A	ROS1 fusion[c]	N/A	CD74-ROS1	No
YU-1081	Crizotinib	NE[f]	0.3	ROS1 fusion[c]	N/A	TPM3-ROS1	No
YU-1082[k]	N/A	N/A	N/A	ROS1 fusion[b]	N/A	SLC34A2-ROS1	No
YU-1083[k]	N/A	N/A	N/A	ROS1 fusion[b]	N/A	SLC34A2-ROS1	No
YU-1085[k]	Crizotinib	PR	4.2	ROS1 fusion[b]	N/A	SLC34A2-ROS1	No

[a]Known patient tumor mutations were detected using PANAMutyper^{TM}R.

[b]Gene fusions were detected using FISH.

[c]Gene fusions were detected using IHC.

[d]Driver mutations were detected using direct sequencing.

[e]Treatment was discontinued because the patient developed nausea.

[f]Not evaluable due to sudden death of the patient.

[g]Treatment was discontinued because cardiac toxicity was observed in the patient.

[h,i,j,k]Models with identical footnotes were established from a same patient.

TKI, tyrosine kinase inhibitor; FISH, fluorescence in situ hybridization; IHC, immuno-histochemistry; PFS, progression-free survival; N/A, not available; PR, partial response; PD, progressive disease; SD, stable disease; NE, not evaluable.

Supplementary Table 5. List of drugs and their targets used for the drug screening in YU-1089 cells.

Drug name	Main target	Combination index calculated by the Bliss Independence model
Gefitinib	EGFR	1.162106
WZ8040	EGFR	1.3012
Pelitinib	EGFR	1.139715
Dacomitinib	EGFR	1.262566
Afatinib	EGFR/HER	1.098068
AZD8931	EGFR/HER	1.196515
Irbinitinib	HER	1.041432
TAE684	ALK	0.950957
Alectinib	ALK	0.973796
Linsitinib	IGF-1R	0.84943
GSK1904529A	IGF-1R	1.037405
AMG-208	c-Met	1.007102
NVP-BVU972	c-Met	0.981581
PD173074	FGFR	0.872689
BGJ398	FGFR	0.963166
AZD4547	FGFR	0.974947
Dovitinib	FGFR	0.926927
PLX-4720	BRAF	1.017424
Vemurafenib	BRAF	1.013732
AZD6244	MEK	0.913459
GSK1120212	MEK	1.035658
SB 203580	p38 MAPK	1.16491
LY2228820	p38 MAPK	1.171036
Ruxolitinib	JAK	0.982608
LY2784544	JAK	0.887484
Tofacitinib	JAK	0.959943
Cyt387	JAK	0.969702
AZD7762	Chk1	1.00908
LY2603618	Chk1	1.048574
SP600125	JNK	1.02319
MK-2206	Akt	0.899676
GSK690693	Akt	0.894215
GDC-0068	Akt	0.93057
Everolimus	mTOR	0.990837
Rapamycin	mTOR	1.055047
BEZ235	mTOR/PI3K	0.973725
GDC-0941	PI3K	0.871631
Buparlisib	PI3K	0.945083
Alpilisib	PI3	1.005288
AS-252424	PI3K	0.933785
NU7441	DNA-PK	0.991935
SB 216763	GSK-3	0.991884
TWS119	GSK-3	1.143602
CHIR-98014	GSK-3	0.990869
Nilotinib	Bcr-Abl	0.979982
WP1130	Bcr-Abl	0.918926
KX2-391	Src	0.984188
Saracatinib	Src	0.871408
SB 431542	TGFBRI	0.981205
SB 525334	TGFBRI	1.091
LDN193189	TGFBRI	1.001958
PF-00562271	FAK	1.029833
NVP-TAE226	FAK	1.09241
Apatinib	VEGFR2	1.022297
SAR131675	VEGFR3	0.949532
BIBF1120	Multikinase	0.843265
Regorafenib	Multikinase	1.108762
OSI-930	Multikinase	1.042681
Tozasertib	Aurora A	0.83032
ZM-447439	Aurora A/B	1.000903
Barasertib	Aurora B	0.857202
SNS-314	Aurora A/B/C	0.858689
AMG 900	Aurora A/B/C	0.906578
Palbociclib	CDK4/6	1.25205
SNS-032	CDK2	1.028809
PHA-793887	CDK2/CDK5/CDK7	1.210334
AZD5438	CDK1/2/9	1.052975
CP 673451	PDGFRa/b	1.14203
Crenolanib	PDGFRa/b	1.067407
BI 2536	Plk1	1.009857

BI6727	Plk1	1.019603
Fosmatinib	Syk	0.992662
IMD 0354	IKKβ	0.887166
TPCA-1	IKKβ	0.97044
KU-60019	ATM	1.027319
Staurosporine	PKCα, PKCγ and PKCη	1.016615
BX-912	PDK1	0.929528
Tie2 kinase inhibitor	Tie2	1.034664
Y-27632	ROCK	0.942434

Combination index value of each drug and olmutinib is shown.
